# Adaptive-Sampling Mamba–Conv-TasNet for Single-Channel Blind Separation of Electromagnetic Signals

**DOI:** 10.3390/s26216844

**Published:** 2026-10-29

**Authors:** Chao Zhou, Youcheng Guo, Xin Li, Ruoning Zhang, Yulin Han, Juncheng Huang, Xuexin Chen

**Affiliations:** 1 Institute of Electronic and Electrical Engineering, Civil Aviation Flight University of China, Chengdu 618307, China; 2 School of Computer Science and Engineering, University of Electronic Science and Technology of China, Chengdu 611731, China

**Keywords:** single-channel blind source separation, electromagnetic signals, adaptive sampling, complex-baseband signals

## Abstract

In passive electromagnetic detection and positioning systems, the complex electromagnetic environment poses significant theoretical challenges to traditional single-channel blind source separation (SCBSS) algorithms. To address the need to balance separation quality, computational efficiency, resource use and generalisation across different detection conditions, we present the Adaptive-Sampling Mamba–Conv-TasNet, which integrates physically constrained front-end routing with local and long-range sequence modelling. A condition encoder estimates each mixture’s occupied bandwidth and characteristic time scale. It then selects the lowest-rate, anti-aliased profile that preserves the detected band. Four parallel convolutional kernels extract multiscale local features. The separator combines Conv-TasNet-based local modelling with Mamba-based long-range sequence modelling. A shared mask head produces two source-specific masks, and profile-specific decoders subsequently reconstruct two complex-baseband sources. Across three test sets and nine evaluated methods, the proposed model attains the lowest mean complex NMSE on every dataset and the lowest cross-domain log-PSD RMSE while maintaining moderate latency, model size and memory demand. These results indicate that the Adaptive-Sampling Mamba–Conv-TasNet offers a favourable overall trade-off among separation quality, computational efficiency, resource use and cross-domain generalisation relative to the evaluated methods.

## 1. Introduction

Modern UAV links typically include telecommand (TC), telemetry (TM) and high-capacity video downlinks. Their RF emissions are concentrated mainly in unlicensed industrial, scientific and medical (ISM) bands, particularly at 2.4 and 5.8 GHz. These dynamic, non-stationary and broadband signals can overlap with urban noise and civilian communications, creating a challenging non-cooperative electromagnetic environment for UAV monitoring and positioning.

Blind source separation aims to recover latent sources from one or more observed mixtures. The single-channel setting is especially relevant to distributed RF/WiFi surveillance for low-altitude UAVs, where deployment density, receiver complexity, power consumption and cost constrain practical system design [1]. Multi-antenna arrays additionally require accurate clock synchronisation and phase calibration. Recovering sources from a single-antenna observation could therefore simplify deployment and extend the coverage of low-cost sensing nodes.

Single-channel RF separation is nevertheless underdetermined and becomes particularly difficult when non-stationary emitters occupy overlapping bands. Classical BSS methods obtain identifiability from assumptions such as statistical independence, non-Gaussianity [2] or time–frequency sparsity [3]. Although these assumptions yield interpretable formulations, they become less informative in strongly overlapping single-channel mixtures. Propagation delay, carrier-frequency offset, phase rotation and receiver noise further suppress the source-specific cues available in one observation. Data-driven time-domain models offer a complementary route by learning discriminative local and long-range patterns directly from complex-baseband samples.

Recent data-driven RF studies have begun to address this underdetermined setting directly. Hinderer adapted supervised time–domain separation to overlapping radar and communication emissions observed by a single receiver [4]. Lee et al. evaluated audio-derived neural separators on co-channel OFDM and showed that domain-informed parameterisation is essential because architectures effective for speech can fail on communication waveforms [5]. The RF Challenge subsequently provided a public benchmark spanning QPSK and OFDM signals with non-Gaussian, non-stationary co-channel interference and evaluated both waveform error and bit-error rate [6]. These studies establish the promise of learned RF separation but mainly target a fixed acquisition configuration, a predefined signal-of-interest/interference role, or a specific waveform family.

Time-domain audio separation provides useful architectural primitives, while recent RF studies have begun to adapt them to complex-baseband signals. Conv-TasNet learns masks in a time–domain representation, and RF-oriented WaveNet variants use learnable dilation and data augmentation to improve separation in dense spectra [7,8]. Self-attention can capture long-range interactions and has been applied directly to RF mixtures [9,10], although its global form retains quadratic sequence complexity, whereas structured state-space models provide a linear-time alternative [11,12]. Recent Conv-Mamba results further indicate that dilated convolution and selective recurrence can be complementary [13]. We use these components as architectural primitives while designing the sampling adaptation and complex-baseband reconstruction specifically for RF signals.

A remaining RF-specific issue is that a convolutional kernel spans a fixed number of samples rather than a fixed physical duration. Thus, changes in receiver sampling rate alter the observed temporal context even when the underlying signal process is unchanged; sampling-frequency-independent convolution studies confirm that this mismatch can materially affect separation networks [14]. Unlike prior data-driven RF approaches, our formulation performs symmetric reconstruction of two complex-baseband sources across acquisition domains and couples the shared separator to an anti-aliasing-constrained profile router. This explicitly adapts the processing time grid while preserving the occupied band, rather than relying on a fixed-rate, waveform-specific front end.

We therefore developed the Adaptive-Sampling Mamba–Conv-TasNet for separating two sources from a single complex-baseband mixture. An anti-aliasing-constrained router combines receiver sampling metadata with estimated spectral occupancy to select a processing profile. A profile-aware multiscale encoder then maps every selected time grid to a common latent dimension. Within the shared separator, dilated TCN blocks model local modulation structure and alternating-direction Mamba scans maintain source identity over longer intervals. This design confines sampling-dependent operations to the front-end and reconstruction filter banks while sharing the principal separator across acquisition conditions.

The study makes three contributions. First, it introduces physically constrained profile routing that selects the lowest feasible processing rate while preserving the detected band. Second, it combines multiscale convolution, dilated TCN blocks and selective state-space recurrence to couple local waveform structure with long-range source continuity. Third, it evaluates the resulting architecture on three RF datasets using waveform, spectral and separation metrics, targeted ablations, cross-domain transfer and a broad input signal-to-noise ratio (SNR) range. Across one within-acquisition-family and two cross-domain test sets, the proposed model attains the lowest mean NMSE among the evaluated methods and maintains comparatively stable SI-SDRi across datasets and SNR levels. The ablations quantify the effects of adaptive sampling, FiLM conditioning and Mamba-based long-range modelling.

## 2. Signal Model and Design Rationale

### 2.1. Complex Baseband Mixture and Mask-Based Separation

At the transmitter, information is mapped to complex symbols *a_i_*[*k*] = *I_i_*[*k*] + *jQ_i_*[*k*], where *I* and *Q* denote the in-phase and quadrature components. After pulse shaping, the complex-baseband signal from source *i* is given by Equation (1).

(1) $$s_{i}\left(t\right)=\underset{k\in \mathbb{Z}}{\sum }a_{i}\left[k\right]g_{i}\left(t-kT_{i}\right)$$

Here, *g_i_*(*t*) is the pulse-shaping waveform and *T_i_* is the symbol interval. Phase-shift keying primarily encodes information in phase, whereas quadrature-amplitude modulation varies both amplitude and phase. Quadrature upconversion produces the real passband waveform in Equation (2).

(2) xiRFt=Resitej2πfc,it=Iitcos2πfc,it−Qitsin2πfc,it

Quadrature downconversion followed by low-pass filtering recovers the complex-baseband observation *y*(*t*) = *I*(*t*) + *jQ*(*t*). Retaining both components preserves amplitude and phase information required for frequency-offset correction, modulation analysis and waveform reconstruction.

At a single receive antenna, multiple propagated signals combine within one observation. For two sources, a discrete complex-baseband mixture of length L is described by Equation (3).

(3) $$y\left[n\right]=\overset{2}{\underset{i=1}{\sum }}A_{i}s_{i}\left[n-\tau_{i}\right]\exp\left\{j\left(\frac{2\pi \Delta f_{i}n}{F_{\mathrm{in}}}+\phi_{i}\right)\right\}+w\left[n\right]$$

Here, F_in_ denotes the physical input sampling rate; *s_i_*[*n*], *A_i_*, *τ_i_*, Δ*f_i_*, and *φ_i_* denote the source waveform, non-negative gain, propagation delay, carrier-frequency offset and initial phase, respectively; and *w*[*n*] denotes receiver noise. The network receives the equivalent two-channel real-valued representation in Equation (4).

(4) x=ReyImy∈R2×L

In the absence of spatial diversity, separation must exploit differences in modulation, spectral occupancy, envelope dynamics and temporal correlations. We therefore use a mask-based separator that maps the mixture to a latent representation *E* and estimates one mask *M_i_* per source. Source reconstruction follows Equation (5).

(5) $$E=\Phi \left(x\right),\;{\hat{E}}_{i}=M_{i}⊙E,\;{\hat{s}}_{i}=\Psi \left({\hat{E}}_{i}\right),\;M_{i}\in {\left[0,1\right]}^{N\times T}$$

Here, *Φ* and *Ψ* denote the encoder and decoder, respectively, and ⊙ denotes element-wise multiplication. Latent masks constrain source estimation to transformations of the encoded mixture and provide a more structured reconstruction space than unconstrained waveform generation.

### 2.2. Time-Domain Convolutional Separation

**Figure 1:**
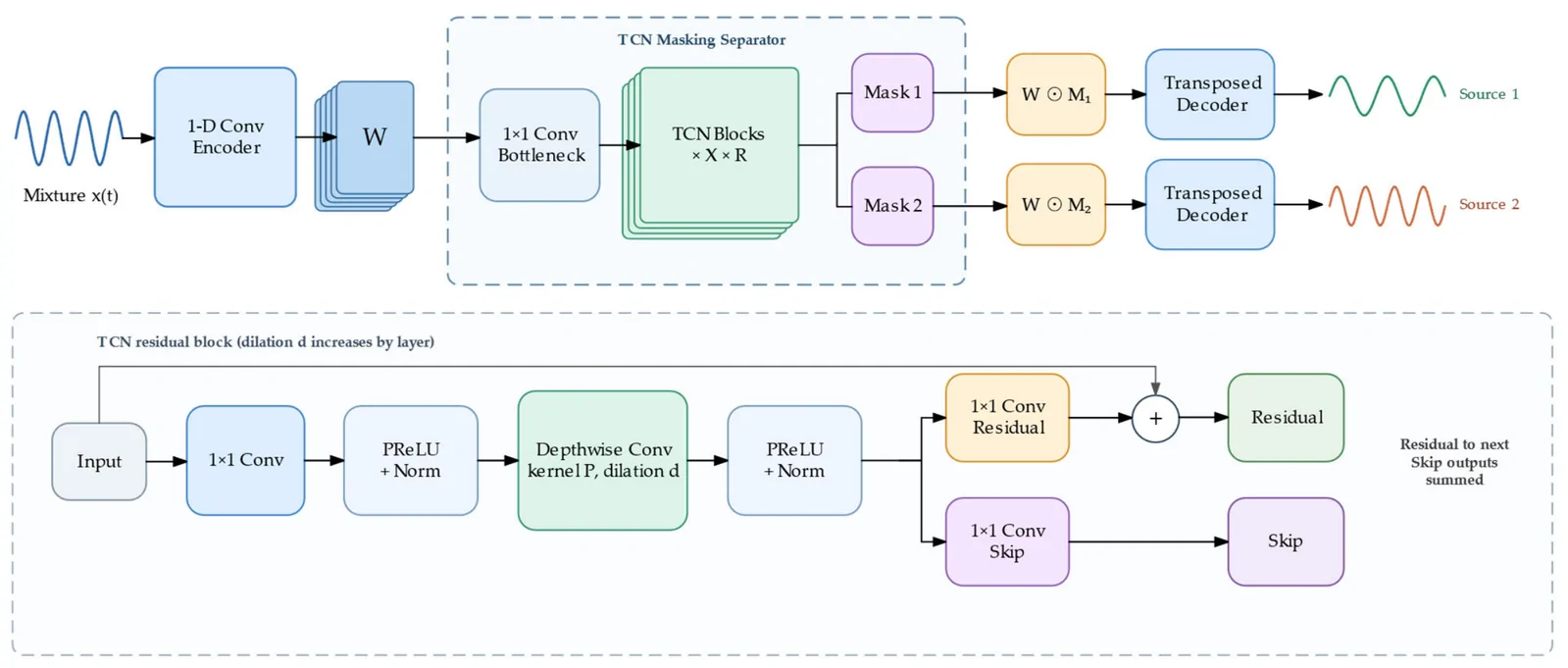
Conv-TasNet architecture.

Conv-TasNet comprises an encoder, a mask-estimation separator and a decoder (Figure 1). The encoder maps mixture *x* to a latent representation *E*; the separator estimates source-specific masks *M_i_*; and the decoder reconstructs each source from the masked latent features.

In Figure 1, the upper panel shows encoding, mask estimation and decoding, and the lower panel shows a dilated TCN block with residual and skip paths. Dilated depthwise convolutions expand the receptive field at moderate computational cost. Residual paths preserve feature propagation, whereas skip paths aggregate evidence across temporal scales. This hierarchy effectively captures local and intermediate-range structure, but dependencies across long signal segments remain mediated indirectly by a finite stack of convolutional blocks.

### 2.3. Selective State Spaces for Long-Range Memory

A discrete state-space model compresses the sequence history into a recurrent hidden state, as expressed in Equation (6).

(6) $$\begin{array}{l} h_{k}={\bar{A}}_{k}h_{k-1}+{\bar{B}}_{k}u_{k}\\ z_{k}=C_{k}h_{k}+Du_{k.} \end{array}$$

Here, *u_k_*, *h_k_*, and *z_k_* denote the current input, hidden state and output, respectively. Mamba makes the state transitions and readouts input dependent, enabling the network to retain separation-relevant history selectively. Unlike global self-attention, this recurrence does not construct an attention matrix whose size grows quadratically with sequence length, making it suitable for long complex-baseband recordings.

**Figure 2:**
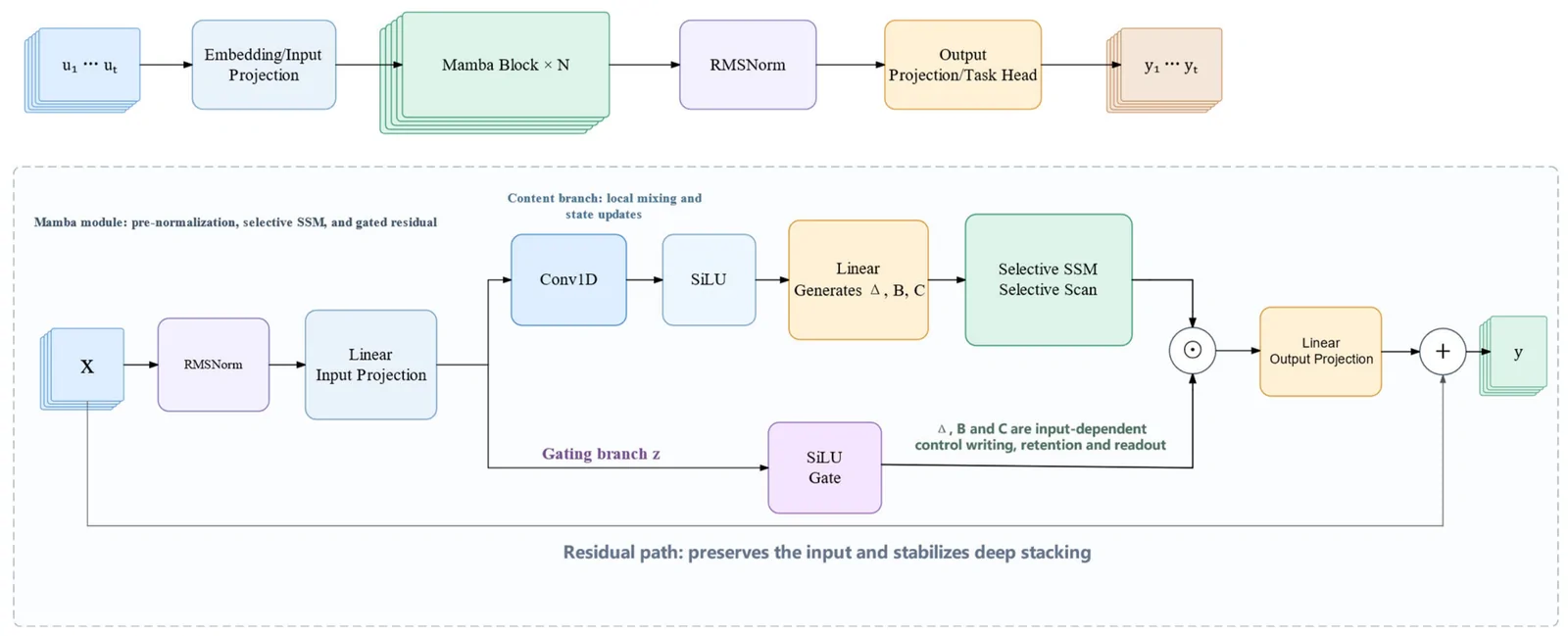
Core architecture of a Mamba block.

As shown in Figure 2, a Mamba block combines pre-normalisation, local convolution, input-dependent parameter generation, selective scanning, gating and a residual connection. The local convolution can encode symbol edges, envelope fluctuations and short-term spectral changes, whereas selective state updates can retain source-related information over longer intervals. These complementary roles motivate placing a Mamba module after each TCN group.

### 2.4. Matching Sampling Scale to the Receptive Field

Signal bandwidth, symbol duration and oscillation scale vary across transmitters and recording systems. Because convolutional kernels are defined in the samples, their physical duration changes with the sampling rate. For a profile rate *F_p_*, kernel length k and local periodic frequency *f*_0_, the temporal coverage and approximate number of periods are defined in Equation (7).

(7) $$T_{k}=\frac{k}{F_{p}},\;N_{k}=f_{0}T_{k}=\frac{kf_{0}}{F_{p}}$$

When *N_k_* < 1, the kernel does not span a complete cycle and may represent phase evolution unreliably. When *N_k_* is substantially larger than one, a single response merges several cycles and can blur transitions, transients and envelope differences. A fixed sampling rate and kernel bank can therefore provide insufficient context, excessive smoothing or redundant computation.

We address this mismatch by selecting a compatible processing profile under an explicit anti-aliasing constraint. Let *F_p_* be the processing sampling rate of candidate profile *p*, and let *r* be the detected half-bandwidth after frequency recentring. For guard factor *η* > 1, the feasible profile set and routing rule are defined in Equation (8).

(8) $$P=\{p:F_{p}/2\geq \mathrm{\eta r}\},\;\;p^{⋆}=\underset{p\in P}{\arg\;\min}F_{p}$$

The router selects a compatible rate that preserves the detected effective band, preferring the lowest feasible processing rate when sampling-rate conversion is applied. This aligns the sequence scale with the shared separator while controlling aliasing and redundant computation.

Sampling profiles also alter the latent time grid seen by the shared separator. For layer *l* with kernel *k_l_*, dilation *d_l_*, and stride *s_l_*, the input-referenced jump *J_l_* and receptive field R_l_ follow Equation (9).

(9) $$\begin{array}{l} J_{0}=1,\;R_{0}=1,\\ J_{l}=J_{l-1}s_{l},\\ R_{l}=R_{l-1}+\left(k_{l}-1\right)d_{l}J_{l-1},\;T_{R}=\frac{R_{l}}{F_{p}} \end{array}$$

The corresponding receptive field has a physical duration determined by the active processing rate. For profile-encoder stride *s_p_*, adjacent latent tokens are separated by Δ*t_p_ = s_p_/F_p_*. The processing rate and encoder stride therefore jointly determine sequence length, the physical TCN receptive field, and the duration represented by each Mamba update. Each profile additionally uses several parallel kernels: short kernels emphasise transients and symbol edges, whereas longer kernels represent periodicity, envelope evolution and slower dynamics.

## 3. Adaptive-Sampling Mamba–Conv-TasNet

Building on Conv-TasNet, we develop the Adaptive-Sampling Mamba–Conv-TasNet, which integrates physical-condition estimation, profile-aware multiscale encoding, hybrid TCN–Mamba separation and multibranch reconstruction. The front end estimates the occupied bandwidth, spectral centre, detector SNR and dominant envelope time scales. It then recentres the spectrum and selects a processing profile whose encoder maps the input to a common latent space. The shared separator alternates dilated TCN groups with forward, backward and forward Mamba scans and uses FiLM conditioning to adapt the hidden features. Profile-specific decoders then reconstruct two complex-baseband sources.

### 3.1. Overall Architecture

**Figure 3:**
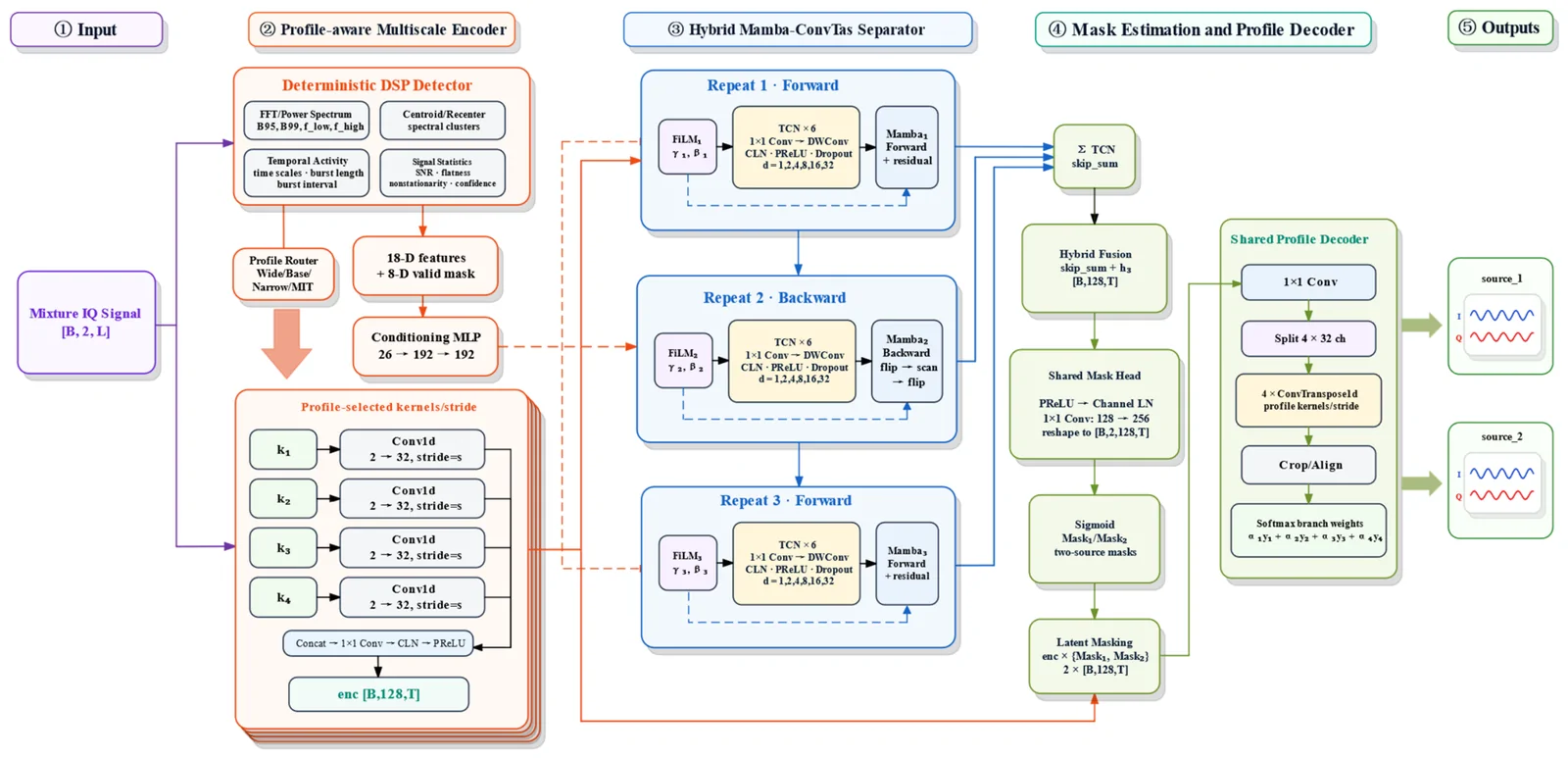
Adaptive-Sampling Mamba–Conv-TasNet architecture.

The proposed network comprises a profile-aware multiscale encoder, an Adaptive-Sampling Mamba–Conv-TasNet separator, a shared mask head that produces two source-specific masks, and profile-specific decoders (Figure 3). The input is a noisy complex-baseband sequence of shape (B, 2, L), and the output consists of two estimated source waveforms, each represented by in-phase and quadrature channels.

The conditioning branch performs profile routing and generates feature-wise linear modulation (FiLM) parameters [15]. All four profile encoders produce 128-dimensional latent features, enabling complete sharing of the downstream separator. The three separator repeats use forward, backward and forward Mamba scans, respectively. The first two contributions to mask estimation are the accumulated TCN skip outputs from the first two repeats, whereas the third contribution is the output of the final Mamba module. Sampling-dependent operations are thus confined to the input and reconstruction filter banks, allowing the shared backbone to learn source structure that transfers across acquisition conditions.

### 3.2. Adaptive Profile Encoder

**Figure 4:**
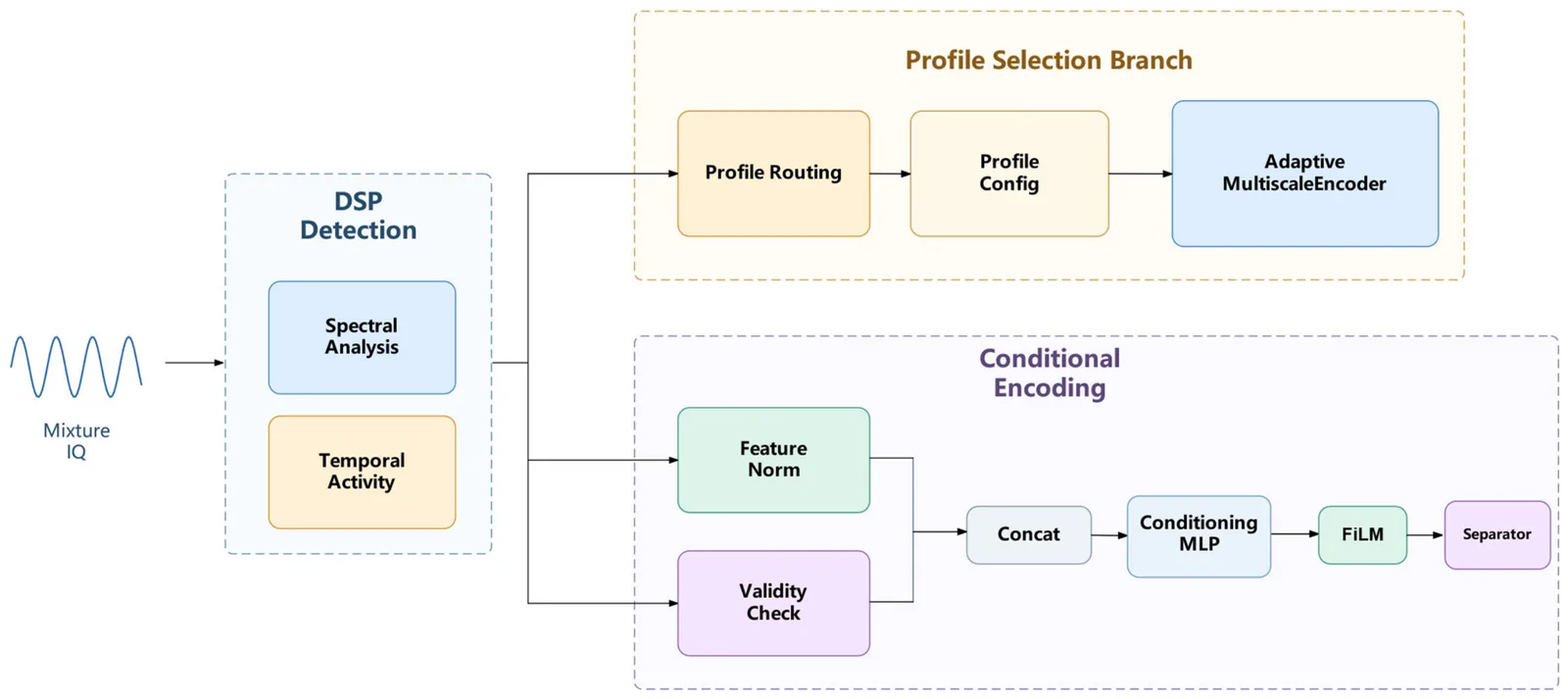
Condition-detection and profile-routing branch.

The adaptive encoder performs physical-condition estimation, profile routing, multiscale convolutional encoding and conditional modulation (Figure 4). Receiver sampling metadata and mixture-band characteristics determine the discrete time scale, while a continuous condition vector modulates channel responses within the shared separator.

Receiver metadata provides the physical sampling rate *F_in_*, which maps sample-domain intervals in the discrete complex-baseband sequence to the physical time and frequency scales used by the detection branch.

For frequency-domain detection, the mixture is framed, windowed and averaged in the power-spectral domain. For frame *ρ*, the centred discrete Fourier transform, mean power spectrum and frequency coordinates are defined in Equation (10).

(10) $$\begin{array}{l} U_{w}=\overset{N-1}{\underset{n=0}{\sum }}|w\left[n\right]{|}^{2},\\ X_{\rho }^{c}\left[k\right]=\overset{N-1}{\underset{n=0}{\sum }}w\left[n\right]x_{\rho }\left[n\right]\exp\left[-j\frac{2\pi }{N}\left(k-\frac{N}{2}\right)n\right],\\ P_{x}\left[k\right]=\frac{1}{RU_{w}}\overset{R}{\underset{\rho =1}{\sum }}|X_{\rho }^{c}\left[k\right]{|}^{2},\\ f_{k}=\left(k-\frac{N}{2}\right)\frac{F_{\mathrm{in}}}{N},\;k=0,1,\ldots ,N-1. \end{array}$$

Here, *R* is the number of frames, *N* is an even transform length, *w*[*n*] is the window and *U_w_* is its energy. The cumulative-power function *C*[*k*] is used to obtain the central 95% and 99% occupied-band intervals:

(11) $$C[k]=\frac{\sum_{j=0}^{k}P_{x}[j]}{\sum_{j=0}^{N-1}P_{x}[j]},\;k_{p}^{-}=\min\{k:C[k]\geq (1-p)/2\},\;\;k_{p}^{+}=\min\{k:C[k]\geq 1-(1-p)/2\},\;f_{p,\mathrm{low}}=f_{k_{p}^{-}},\;\;f_{p,\mathrm{high}}=f_{k_{p}^{+}},\;\;B_{p}=f_{p,\mathrm{high}}-f_{p,\mathrm{low}},\;\;p\in \{0.95,0.99\}.$$

Separately, a 20th-percentile spectral noise floor and a 6 dB margin identify contiguous active clusters. Retained clusters determine the spectral centre *f_c_*, whereas the recentred 99% cumulative-power interval defines the routing bounds. The recentred waveform and routing radius are defined in Equation (12); the cluster-retention and centre-selection rules are summarised in Table A4.

(12) $$x_{c}[n]=x[n]\exp\left(-j\frac{2\pi f_{c}n}{F_{\mathrm{in}}}\right),\;f_{\mathrm{low}}=f_{99,\mathrm{low}}-f_{c},\;\;f_{\mathrm{high}}=f_{99,\mathrm{high}}-f_{c},\;r=\max\left(\left|f_{\mathrm{low}}|,|f_{\mathrm{high}}\right|\right).$$

Together, *F_in_* and *r* determine the feasible profile set in Equation (8). The centred waveform is then converted to the selected profile rate *F_p_*:

(13) $$x_{p}=R(x_{c};F_{\mathrm{in}}\to F_{p})$$

Here, ℛ denotes the shared polyphase sampling-rate-conversion operator from *F_in_* to *F_p_*. Its Kaiser-window FIR response preserves the detected band within the selected Nyquist limit. The four profiles follow the same processing flow and differ only in the parameter settings reported in Table 1.

The time-domain detector divides the complex envelope *a*[*n*] = |*x_c_*[*n*]| into *M* non-overlapping blocks of *K* samples and computes the mean envelope energy *e*[*m*] in each block. Activity and burst statistics are derived from this sequence, while its normalised autocorrelation provides the dominant time scales:

(14) $$e[m]=\frac{1}{K}\sum_{n=0}^{K-1}{\left|x_{c}[\mathrm{mK}+n]\right|}^{2},\;\;\bar{e}=\frac{1}{M}\sum_{m=0}^{M-1}e[m],\;\rho_{e}[l]=\frac{\sum_{m=l}^{M-1}\left(e[m]-\bar{e}\right)(e[m-l]-\bar{e})}{\sum_{m=0}^{M-1}{\left(e[m]-\bar{e}\right)}^{2}},\;\tau_{q}=\frac{l_{q}K}{F_{\mathrm{in}}},\;\;q=1,2,3.$$

Up to three nonzero lags with *ρ_e_*[*ℓ*] > 0.1 are retained and converted to physical time. Frequency bounds, occupied bandwidth, dominant time scale, non-stationarity and confidence are normalised and concatenated with a validity mask. The mask identifies missing or unreliable measurements and allows the conditioning network to distinguish them from valid feature values. A multilayer perceptron maps the resulting 26-dimensional condition vector to 192 dimensions and generates FiLM parameters. Discrete profile routing governs filtering, resampling and encoding, whereas FiLM provides continuous channel-wise adaptation within the shared backbone.

The profile parameters were determined through a separate configuration-selection procedure conducted before formal model training. The candidate space included both detector-routing parameters and encoder parameters. Specifically, the bandwidth expansion coefficient *η* was varied from 1.00 to 2.00 in increments of 0.05, the noise-floor quantile *q* from 0.05 to 0.50 in increments of 0.05, and the detection margin *Γ* from 0 to 10 dB in increments of 0.5 dB. The four-branch kernel bank was parameterised by a base kernel length *k*_0_, where *k*_0_ ∈ {2,4,8}, giving 𝒦(*k*_0_) = [*k*_0_, 2*k*_0_, 4*k*_0_, 8*k*_0_]. The encoder stride *s* was selected from {1,2,4,8}, subject to *s* ≤ *k*_0_. Thus, both the kernel lengths and stride were treated as search variables, while the octave-spaced kernel structure restricted the search to computationally meaningful multiscale configurations.

At each of the four configured processing rates (312.5, 156.25, 78.125, and 25 MHz), 50 candidate configurations were sampled for each input under identical data conditions. After each round, the lowest-performing 5% were discarded until one configuration remained. The selected configurations define P1–P4 and were fixed before the formal 16,000-update training and evaluation. Their encoder parameters are reported in Table 1, and the shared detector and routing settings are summarised in Table A4.

Each profile contains four parallel one-dimensional convolutions. Explicit asymmetric SAME padding aligns token counts across branches. The branch features are concatenated and fused using a 1 × 1 convolution, channel normalisation and PReLU. Because every profile produces a 128-channel latent representation, a single TCN–Mamba separator can process all routes. The digital-signal-processing detector, profile bank and FiLM module respectively provide physical-scale estimation, discrete representation selection and continuous channel-wise adaptation.

### 3.3. Adaptive-Sampling Mamba–Conv-TasNet Separator

**Figure 5:**
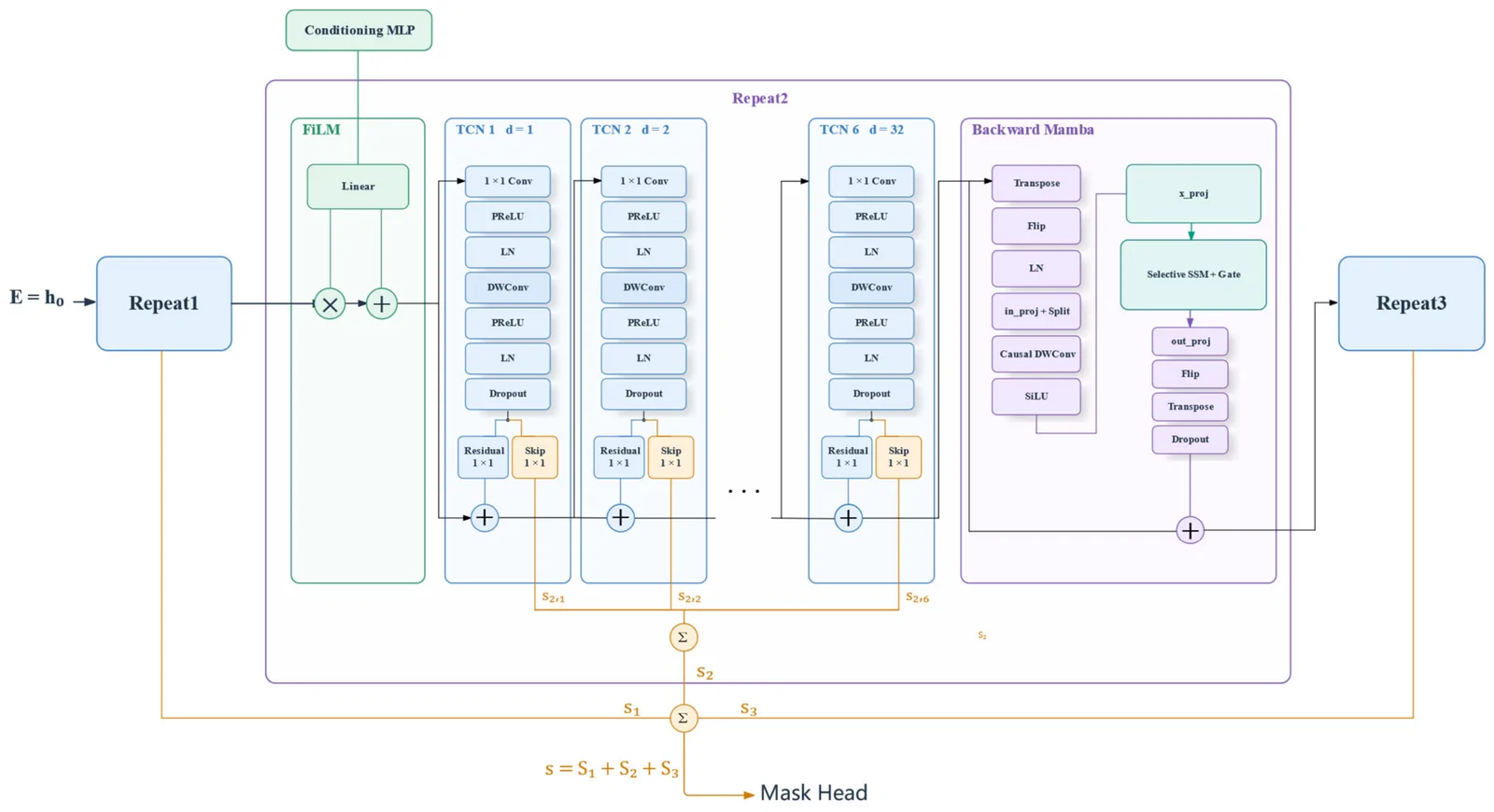
Internal structure of the Adaptive-Sampling Mamba–Conv-TasNet separator.

The separator contains three repeated units. Within each unit, the 192-dimensional condition vector is mapped to channel-wise FiLM scale and bias terms. These parameters are initialised to approximate an identity mapping, allowing the network to learn profile- and signal-dependent channel reweighting during training. Figure 5 depicts the second repeat; the first and third repeats use the same topology without sequence reversal.

Each unit first applies six TCN blocks with dilations of 1, 2, 4, 8, 16 and 32. Each block contains a 1 × 1 bottleneck projection followed by a depthwise dilated convolution with kernel length 3. Channel normalisation, PReLU and dropout are then applied, after which the block produces residual and skip outputs. The bottleneck contains 96 channels, and the dropout probability is 0.05.

A Mamba module follows each TCN group. Its model dimension is 128, state dimension is 16, local-convolution width is 4 and expansion factor is 2. The three repeats scan in forward, backward and forward directions, respectively. The middle repeat reverses the sequence before selective scanning and restores the original order afterwards. This arrangement introduces backward context while retaining predominantly forward information flow at the beginning and end of the separator. For mask estimation, the accumulated TCN skip outputs from the first and second repeats form S_1_ and S_2_, while the output of the final Mamba module forms S_3_. Their sum *S* = *S*_1_ + *S*_2_ + *S*_3_ is passed to the mask head, ensuring that the final long-range representation contributes directly to source-mask estimation.

### 3.4. Mask Estimation and Profile-Specific Decoding

The mask head comprises PReLU, channel normalisation and a 1 × 1 convolution. It maps the 128-channel separator output to two 128-channel masks, each constrained to [0, 1] by a sigmoid. Element-wise multiplication with the encoded mixture produces a source-specific latent representation for each output.

For decoding, each masked representation is divided into four 32-channel branches. Transposed convolutions matched to the kernels and stride of the active profile restore temporal resolution. Each branch is cropped using the left and right padding recorded during encoding, and learned softmax weights combine the four aligned reconstructions. The final output contains two complex-baseband sources, each represented by I and Q channels.

### 3.5. Mixture Synthesis and Training

Training data were drawn from the MPACT Lab Drone Remote Controller RF Signal Dataset recorded at North Carolina State University [16,17]. We used four transmitter classes: DJI Inspire 1 Pro, DJI Matrice 100 and two DJI Matrice 600 controllers, SZ DJI Technology Co., Ltd., Shenzhen, China. Before mixture synthesis, each source waveform *s_i_*, originally sampled at *F*_src*,i*_, was converted to the common physical input rate *F*_in_ on a shared physical time grid, as expressed in Equation (15). Frequency alignment was applied consistently to both sources. The resulting mixture was then routed through one of the four configured profiles using the bandwidth-preservation rule in Equation (8), where *F_p_* denotes the processing rate of the selected profile.

(15) $${\tilde{s}}_{i}=R(s_{i};F_{\mathrm{src},i}\to F_{\mathrm{in}}),\;\;i\in \{1,2\}$$

After sampling-rate conversion, source powers were adjusted to a randomly selected signal-to-interference ratio (SIR). Measured noise, synthetic Gaussian noise (white or coloured), or impulsive noise was then added at the target SNR. Measured noise was converted to the selected sampling grid, whereas synthetic noise was generated directly at that rate. If *P*(*z*) denotes mean complex-sequence power and v the initial noise realisation, the mixture and targets are formed according to Equation (16).

(16) $$\begin{array}{l} P\left(z\right)=\frac{1}{L}\overset{L-1}{\underset{n=0}{\sum }}|z\left[n\right]{|}^{2},\\ a=\sqrt{\frac{P\left({\tilde{s}}_{1}\right)}{P\left({\tilde{s}}_{2}\right)10^{{\mathrm{SIR}}_{\mathrm{dB}}/10}}},\;u={\tilde{s}}_{1}+a{\tilde{s}}_{2},\\ b=\sqrt{\frac{P\left(u\right)}{P\left(v\right)10^{{\mathrm{SNR}}_{\mathrm{dB}}/10}}},\;x=\frac{u+\mathrm{bv}}{c},\\ \left(t_{1},t_{2}\right)=\left(\frac{{\tilde{s}}_{1}}{c},\frac{a{\tilde{s}}_{2}}{c}\right). \end{array}$$

Here, *L* is the sequence length; a scales the second resampled source ${\tilde{s}}_{2}$ to the sampled SIR; *u* is the resulting clean mixture; *b* scales the noise realisation *v* to the sampled SNR; *x* is the normalised noisy input; and *t*_1_ and *t*_2_ are the corresponding normalised supervision targets. The common divisor *c* is a robust amplitude scale computed from the noisy mixture, with *b* = 0 for noise-free examples. The noisy input, clean mixture and supervision targets therefore share this scale, preserving source-amplitude ratios that would otherwise be distorted by independent normalisation.

Because the two output channels have no intrinsic ordering, we use permutation-invariant training (PIT) [18]. Both direct and swapped pairings are evaluated, and the pairing with the lower total loss defines the assignment. The pairwise objective combines complex-baseband waveform error, scale-invariant signal-to-noise ratio (SI-SNR) and a multiresolution short-time Fourier transform (MR-STFT) loss [19], as expressed in Equation (17).

(17) $$\begin{array}{l} l_{\mathrm{ij}}=\lambda_{\mathrm{IQ}}L_{\mathrm{IQ}}\left({\hat{s}}_{i},t_{j}\right)+\lambda_{\mathrm{SI}}L_{\mathrm{SI}-\mathrm{SNR}}\left({\hat{s}}_{i},t_{j}\right)+\lambda_{\mathrm{TF}}L_{\mathrm{MR}-\mathrm{STFT}}\left({\hat{s}}_{i},t_{j}\right),\\ L_{\mathrm{PIT}}=\underset{\pi \in P_{2}}{\min}\overset{2}{\underset{i=1}{\sum }}l_{i,\pi \left(i\right)} \end{array}$$

Two auxiliary terms are added after PIT. The mixture-consistency loss, *L_mix_*, is the batch-mean relative complex-baseband mean-squared error between the sum of the two estimates and the clean mixture used to generate the training example [20]. For each training example *b*, the implementation computes *r_b_*, the magnitude of the normalised complex inner product between the two predicted complex-baseband sequences. The inner product conjugates the first output, and its denominator is the product of the two L2 norms with a numerical floor of 10^−12^; hence, 0 ≤ *r_b_* ≤ 1. The output-collapse loss is $L_{\mathrm{col}}\;=\;\frac{1}{B}\;\sum_{b}\;\max(r_{b}\;-\;0.98,\;0)$, where B is the batch size. It is inactive unless the two estimates are almost collinear and therefore specifically discourages both output channels from reconstructing the same source; low output energy alone is not penalised by this term. The overall objective is:

(18) $$L=L_{\mathrm{PIT}}+\lambda_{\mathrm{mix}}L_{\mathrm{mix}}+\lambda_{\mathrm{col}}L_{\mathrm{col}}$$

Here, the PIT term is the permutation-selected pairwise objective in Equation (17), while *λ*_mix_ and *λ*_col_ are the weights of the mixture-consistency and output-collapse terms, respectively.

All experiments were conducted under Ubuntu on WSL2 using an NVIDIA GeForce RTX 4060 Laptop GPU with 8 GB of VRAM. The software environment comprised Python 3.10.12, PyTorch 2.7.0+cu126, CUDA 12.6, cuDNN 9.5.1, Mamba-SSM 2.3.2.post1 and causal-conv1d 1.6.2.post1. The proposed model was trained for 16,000 parameter updates with AdamW. The initial and minimum learning rates were 1 × 10^−4^ and 1 × 10^−6^, respectively. Weight decay was 1 × 10^−4^, momentum coefficients were (0.9, 0.98) and the gradient-clipping threshold was 5. Training used BF16 mixed precision and four-step gradient accumulation. Micro-batch sizes across the four curriculum stages were 1, 2, 2 and 4, with corresponding proportions of 10%, 20%, 50% and 20%. Loss weights for complex-baseband reconstruction, SI-SNR, multiresolution STFT, mixture consistency and output-collapse loss were 1.00, 0.10, 0.05, 0.05 and 0.01, respectively.

## 4. Experiments

### 4.1. Test Sets and Comparator Models

We evaluated the models on three test sets with progressively larger shifts in acquisition conditions. D1 contained DJI Phantom 3 and DJI Phantom 4 Pro controller recordings from the MPACT dataset. These recordings belong to the training acquisition family but represent transmitter models that were not used for training. D2 contained 40 MHz complex-baseband recordings from DroneMMSet, a multimodal dataset collected for drone monitoring in complex urban environments [21]. D3 used RF recordings from the MIT RF Challenge to assess transfer across communication signals and acquisition conditions [6].

Every evaluation example contained two distinct sources synthesised using the same mixture procedure as the training data. SIR values were sampled uniformly between −5 and 5 dB. Input SNRs were evaluated at −10, −6, −2, 2, 6, 10, 14, 18, 22, 26 and 30 dB. For each test set, 100 mixtures were generated at every SNR, yielding 1100 examples per set and 3300 examples overall. This balanced design reduces sampling variability and supports direct comparison across datasets and operating conditions.

The learned comparators comprised a Transformer-based separator, Wave-U-Net [22], DPRNN-TasNet (DPRNN) [23], and three ablations that removed Mamba, adaptive sampling, or FiLM conditioning. The Transformer uses stacked self-attention and feed-forward blocks to model global dependencies across the full input sequence. Wave-U-Net is a multiscale one-dimensional encoder–decoder that separates sources directly in the time domain, whereas DPRNN partitions a long sequence into overlapping chunks and alternates intra- and inter-chunk recurrent modelling. The non-learning comparators were EEMD–ICA, which combines noise-assisted ensemble empirical mode decomposition [24] with independent component analysis (ICA) [2], and VMD–ICA, which combines variational mode decomposition [25] with ICA. All learned models used identical data partitions and dynamic mixture-generation procedures. Every method was evaluated on the same waveform mixtures with the same output matching and metrics; architecture-specific front-end operations were treated as part of each model. Detailed dataset, architecture, training and environment configurations are provided in Appendix A.

### 4.2. Evaluation Metrics

Because the two source signals have no intrinsic ordering, a well-separated output pair may still be assigned to the opposite reference order during evaluation. To avoid this mismatch, we selected the output permutation that maximised scale-invariant signal-to-distortion ratio (SI-SDR) for each example and used it for all remaining metrics. For mean-centred sequences and complex projection coefficient (α), SI-SDR and the optimal permutation are defined in Equation (19).

(19) $$\begin{array}{l} \alpha =\frac{{\tilde{s}}^{H}\tilde{\hat{s}}}{{\tilde{s}}^{H}\tilde{s}+\varepsilon },\\ \mathrm{SI}-\mathrm{SDR}\left(\hat{s},s\right)=10\log_{10}\frac{{∥\alpha \tilde{s}∥}_{2}^{2}+\varepsilon }{{∥\tilde{\hat{s}}-\alpha \tilde{s}∥}_{2}^{2}+\varepsilon },\\ \pi^{*}=\underset{\pi \in P_{2}}{\mathrm{argmax}}\frac{1}{2}\overset{2}{\underset{k=1}{\sum }}\mathrm{SI}-\mathrm{SDR}\left({\hat{s}}_{\pi \left(k\right)},s_{k}\right) \end{array}$$

SI-SDR improvement (SI-SDRi), measured relative to the noisy mixture, quantifies the net separation gain according to Equation (20).

(20) $$\mathrm{SI}-\mathrm{SDRi}=\frac{1}{2}\overset{2}{\underset{k=1}{\sum }}\left[\mathrm{SI}-\mathrm{SDR}\left({\hat{s}}_{\pi^{*}\left(k\right)},s_{k}\right)-\mathrm{SI}-\mathrm{SDR}\left(x,s_{k}\right)\right]$$

Complex normalised mean-squared error (NMSE) directly compares the PIT-matched complex-baseband waveform without correcting source-specific gain or constant phase:

(21) $${\mathrm{NMSE}}_{\mathrm{dB}}=\frac{1}{2}\overset{2}{\underset{k=1}{\sum }}10\log_{10}\left(\frac{{∥{\hat{s}}_{\pi^{*}\left(k\right)}-s_{k}∥}_{2}^{2}+\varepsilon }{{∥s_{k}∥}_{2}^{2}+\varepsilon }\right)$$

Normalised log-power-spectral-density (log-PSD) root-mean-square error measures distortion of spectral shape. The mean Hann-windowed spectrum is normalised to unit energy and converted to decibels:

(22) $$\begin{array}{l} {\tilde{P}}_{z}\left[k\right]=\frac{P_{z}\left[k\right]}{\sum_{l=1}^{K}P_{z}\left[l\right]+\varepsilon },\\ L_{z}\left[k\right]=10\log_{10}\left(\max\left\{{\tilde{P}}_{z}\left[k\right],10^{-12}\right\}\right),\\ e_{r}=\sqrt{\frac{1}{K}\overset{K}{\underset{k=1}{\sum }}{\left(L_{{\hat{s}}_{\pi^{*}\left(r\right)}}\left[k\right]-L_{s_{r}}\left[k\right]\right)}^{2}},\\ {\mathrm{RMSE}}_{\mathrm{PSD}}=\frac{1}{2}\overset{2}{\underset{r=1}{\sum }}e_{r}. \end{array}$$

Here, the tilde denotes unit-energy spectral normalisation, K is the number of frequency bins, and ε is a small constant added to the denominator during spectral normalisation to ensure numerical stability, whereas 10^−12^ is the floor applied before taking the logarithm. *L_z_*[*k*] is the resulting normalised log-PSD, and *e_r_* is the log-PSD RMSE for source r after optimal permutation matching. A higher SI-SDRi denotes a larger separation gain over the noisy mixture, whereas lower complex NMSE and log-PSD RMSE denote more accurate complex-waveform and spectral-shape reconstruction, respectively. In Table 2, latency measures the mean end-to-end processing time. The real-time factor (RTF) is the mean latency divided by the physical input duration *L/F_in_*, where *L* = 2048 is the input length and *F*_in_ is the physical input sampling rate of the test example; lower values are better, and *RTF* < 1 indicates real-time operation. The trainable-parameter count reflects model size, while peak CUDA and CPU memory describe device- and host-memory demand. Peak CUDA memory is reported only for GPU-executed neural models; EEMD–ICA and VMD–ICA are marked as “CPU only” because they were executed exclusively on the CPU. For these conventional methods, the trainable-parameter column is zero because decomposition coefficients and demixing matrices are estimated at run time; their computational demand is represented by latency and CPU memory. Unless otherwise stated, reconstruction results are reported as the mean ± standard deviation across test examples.

### 4.3. Quantitative Results and SNR Dependence

Table 2 shows that the Adaptive-Sampling Mamba–Conv-TasNet achieved the lowest mean NMSE on D1, D2 and D3, with values of −25.08, −22.66 and −20.27 dB, respectively. It also achieved the lowest log-PSD RMSE on D2 and D3. The corresponding values of 6.38 and 6.26 dB were 28.4% and 32.5% lower than those of the strongest comparator on each dataset. On D1, the Transformer produced a marginally lower log-PSD RMSE than the proposed model, 5.74 versus 5.77 dB, corresponding to a 0.5% reduction. However, its NMSE degraded to −2.98 dB on D2 and −1.49 dB on D3, compared with −22.66 and −20.27 dB for the proposed model. Wave-U-Net was 3.2% faster on D1, with latencies of 5.60 and 5.788 ms, respectively. However, Wave-U-Net required 489.9% more trainable parameters and 105.1% more peak CUDA memory. These results indicate that the Adaptive-Sampling Mamba–Conv-TasNet offers a favourable overall trade-off among separation quality, computational efficiency and resource use under the evaluated conditions. Removing FiLM saved 190,848 parameters but worsened NMSE by 10.87–12.40 dB and increased log-PSD RMSE by 2.26–5.60 dB, indicating that continuous conditioning provides substantial accuracy gains for a modest parameter cost.

**Figure 6:**
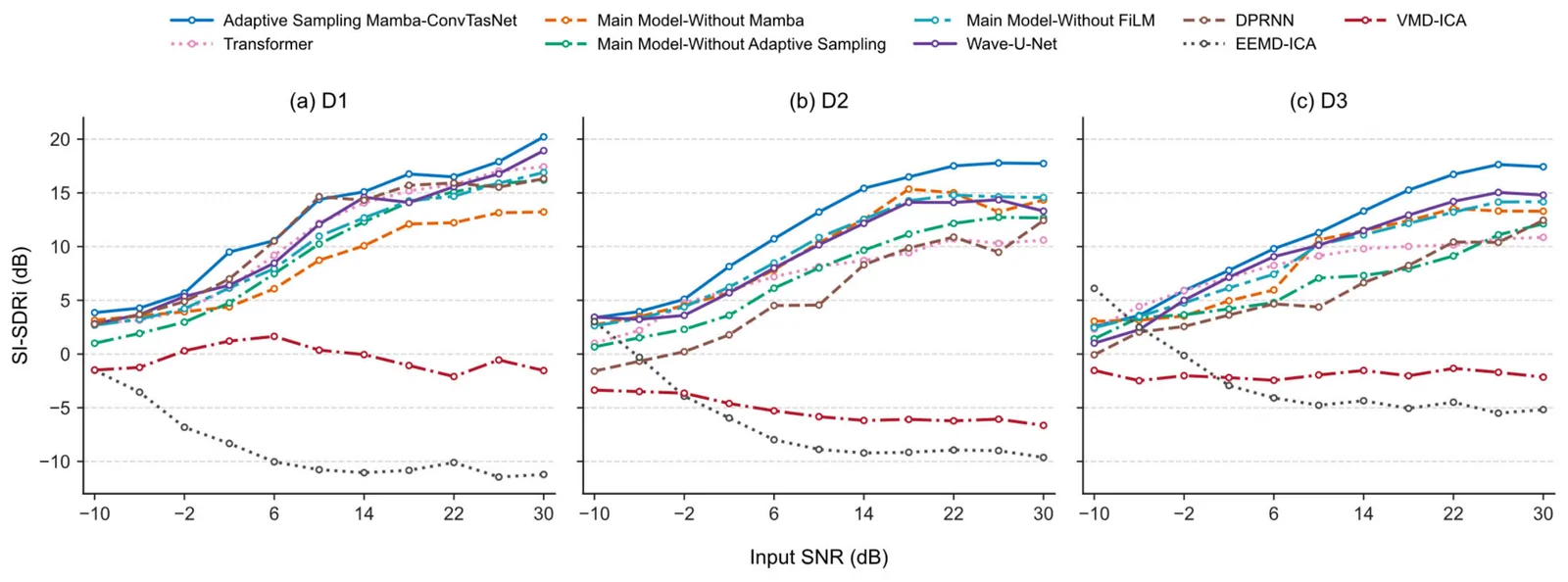
Mean SI-SDRi of the nine models at different input SNRs on D1–D3. (**a**) D1: MPACT dataset; (**b**) D2: DroneMMSet dataset; (**c**) D3: MIT RF Challenge.

Figure 6 shows how SI-SDRi varies with input SNR for each evaluated model. Differences are compressed at the lowest SNRs because noise masks source-specific structure. From intermediate SNR onward, the Adaptive-Sampling Mamba–Conv-TasNet shows comparatively stable performance across datasets and ranks among the highest in SI-SDRi at most operating points. By contrast, the decomposition-based ICA methods frequently yield little or negative improvement.

Wave-U-Net is competitive on D1 and D2 but reaches a lower performance ceiling on D3, indicating weaker transfer from drone-controller signals to the more heterogeneous signal types in D3. The Transformer likewise drops sharply from D1 to D2 and D3 and saturates at a substantially lower SI-SDRi, suggesting weaker cross-domain transfer under the tested conditions. The no-FiLM ablation follows the overall SNR-dependent trend of the full model but remains lower, particularly on D2 and D3, showing that discrete profile routing alone does not recover the benefit of continuous feature conditioning. By contrast, the proposed model preserves a consistent curve shape across all three datasets, supporting the combined value of adaptive physical-scale matching, FiLM conditioning and long-range sequence modelling.

### 4.4. Time- and Frequency-Domain Reconstruction

**Figure 7:**
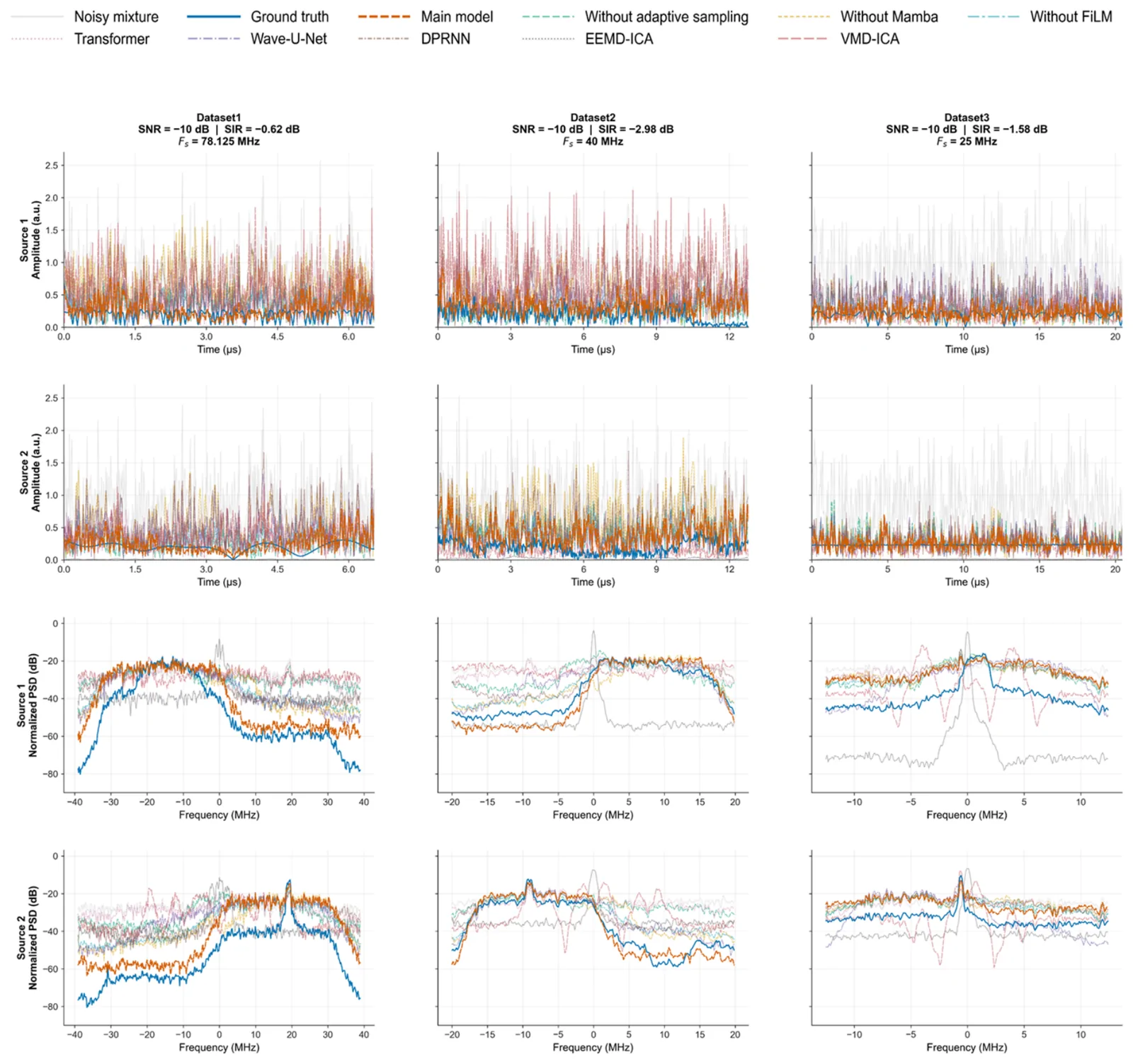
Time- and frequency-domain outputs of all models at an input SNR of −10 dB.

**Figure 8:**
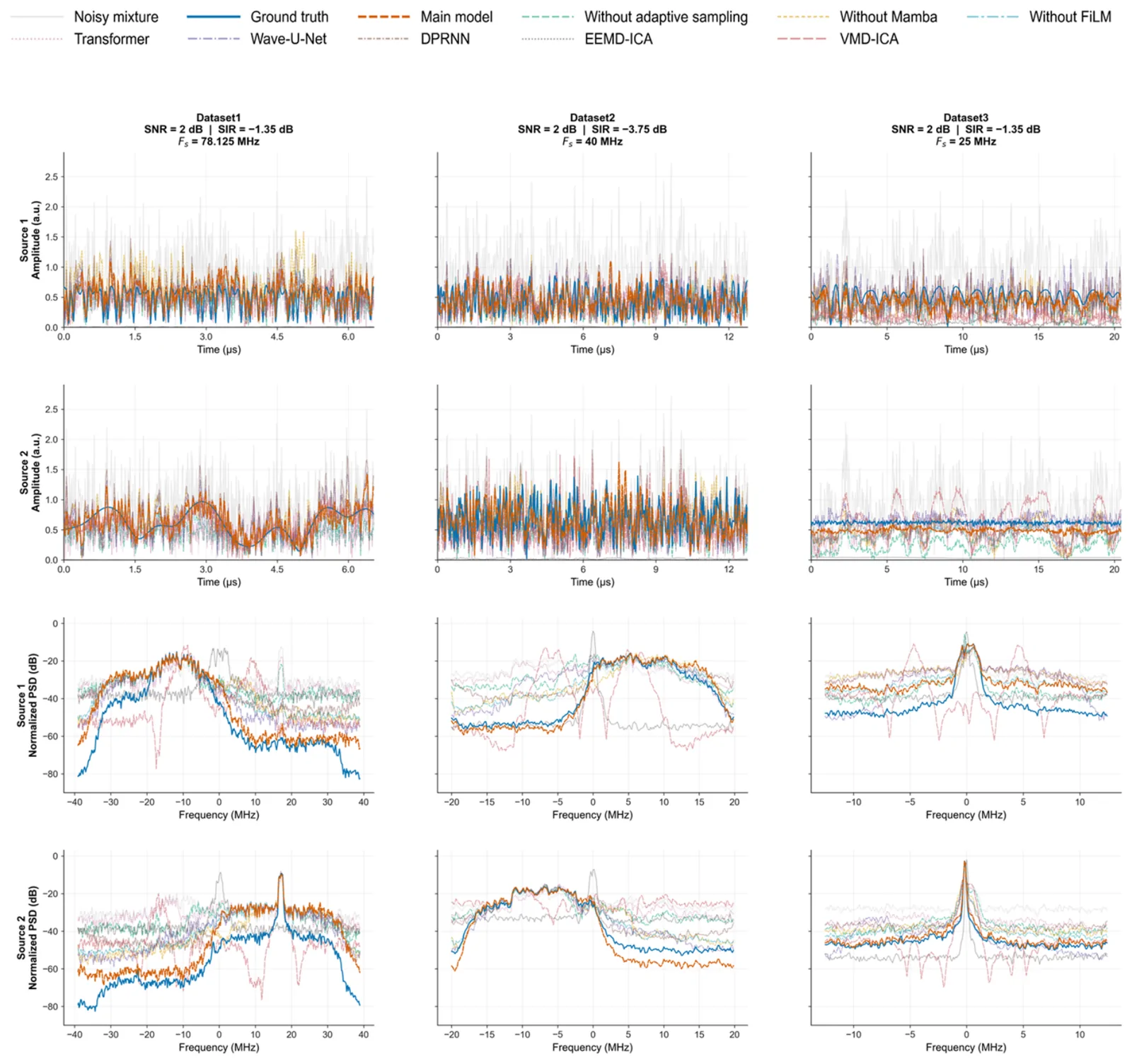
Time- and frequency-domain outputs of all models at an input SNR of 2 dB.

**Figure 9:**
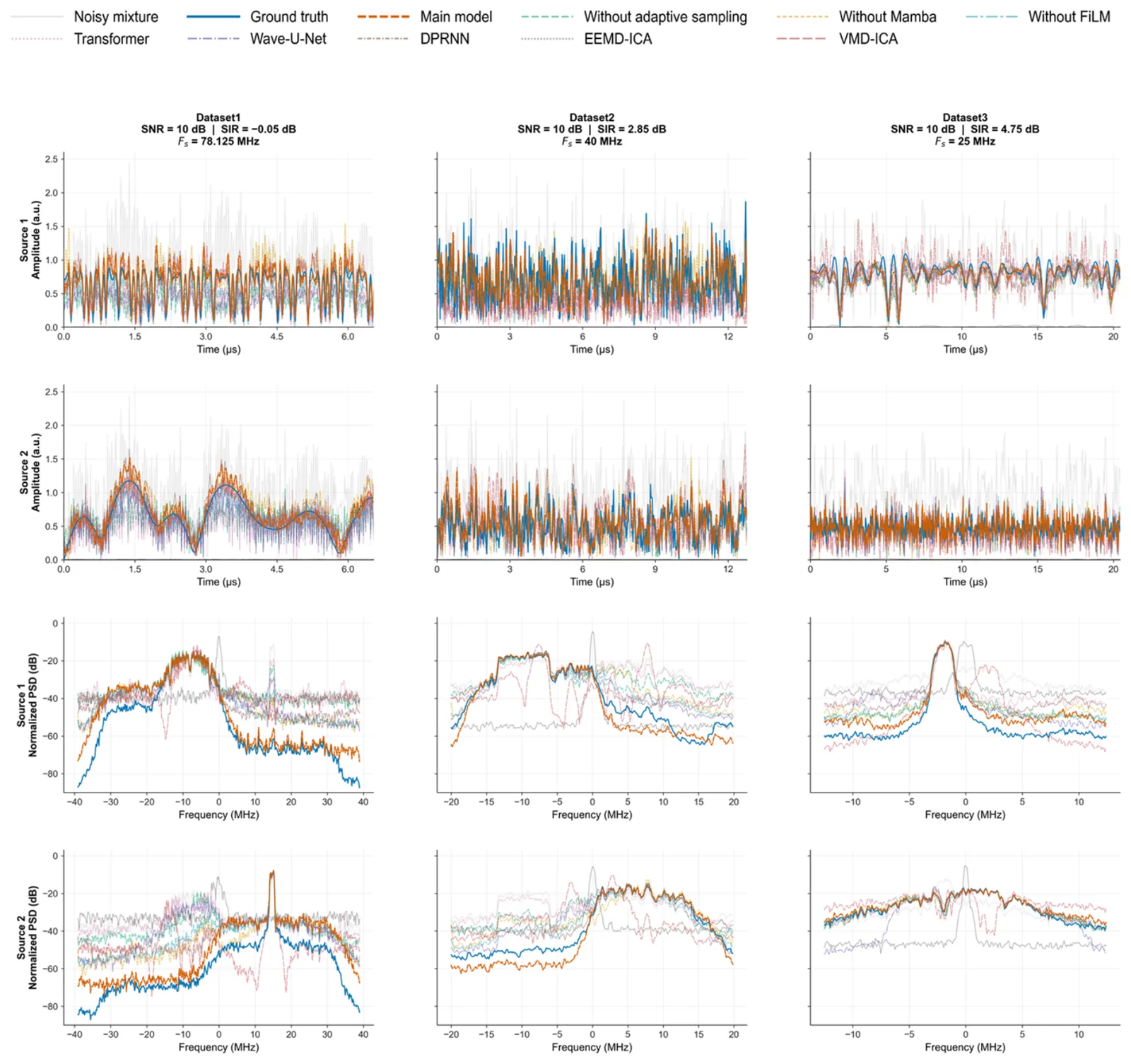
Time- and frequency-domain outputs of all models at an input SNR of 10 dB.

**Figure 10:**
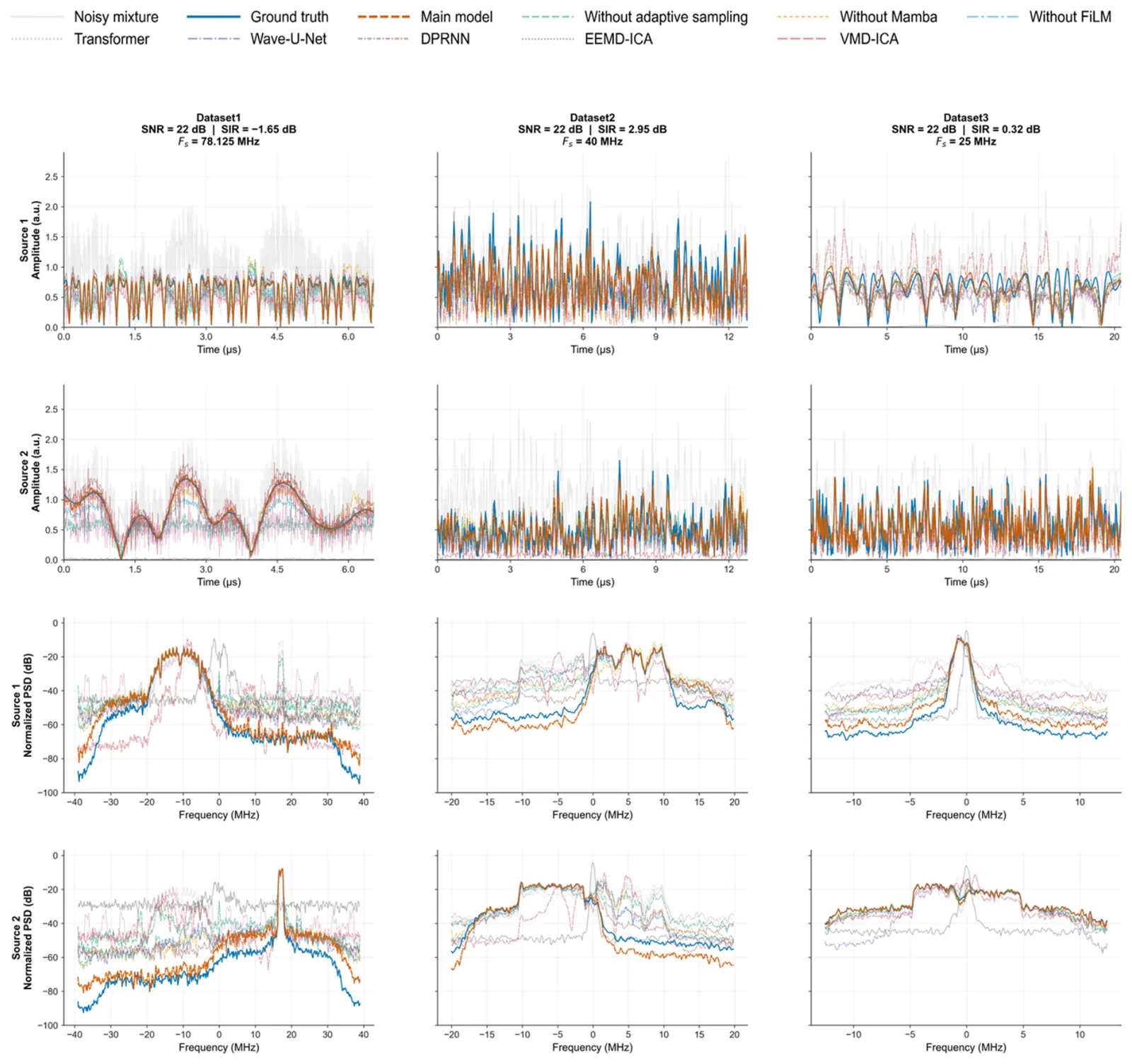
Time- and frequency-domain outputs of all models at an input SNR of 22 dB.

**Figure 11:**
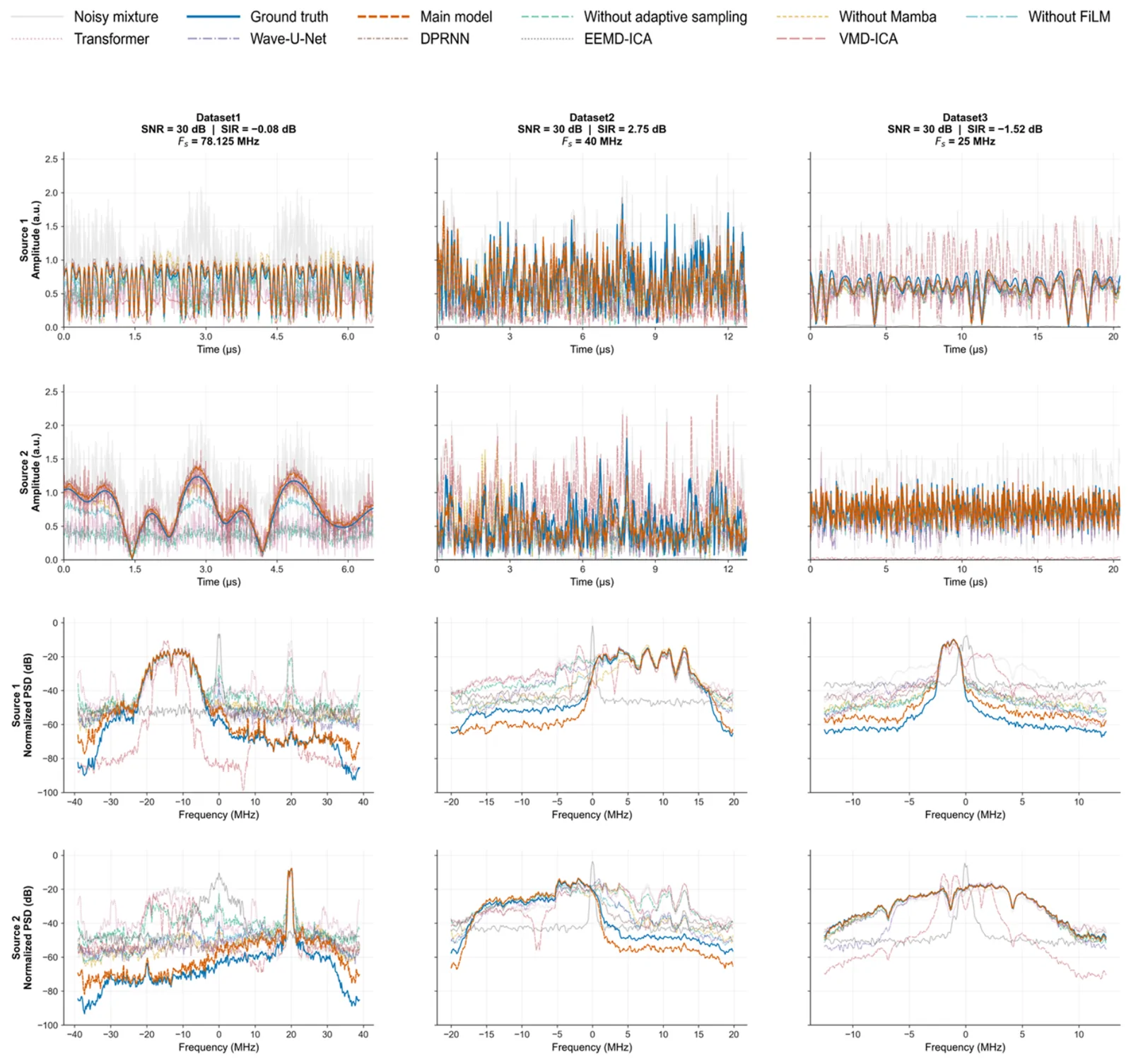
Time- and frequency-domain outputs of all models at an input SNR of 30 dB.

Figure 7, Figure 8, Figure 9, Figure 10 and Figure 11 compare the outputs of all nine models on D1–D3 at five representative input SNRs (−10, 2, 10, 22 and 30 dB). For each dataset, the first two rows show time-domain amplitudes over samples 768–1279, and the last two rows show normalised power spectral densities calculated from the complete 2048-sample complex-baseband sequences using a Hann window and zero-padding to 8192 points.

Across the five SNR levels, the estimates progressively converge towards the ground-truth temporal envelopes and spectral shapes as noise decreases. At −10 dB, all methods show larger amplitude fluctuations, elevated spectral floors and weaker recovery of low-energy components. From 10 to 30 dB, the learned separators recover the dominant waveform structure and occupied bands more clearly, although errors remain around sharp transients and weak spectral sidelobes.

In the examples shown, the Adaptive-Sampling Mamba–Conv-TasNet exhibits comparatively consistent agreement with the references, particularly for D2 and D3. The largest discrepancies for the comparator methods appear as broadband residuals, mode mixing or suppression of one source, rather than a uniform loss of all structure. These examples also suggest that the proposed model is comparatively less sensitive to changes in signal type and acquisition condition.

### 4.5. Effect of Bandwidth-Estimation Error on Profile Selection and Model Output

To isolate sensitivity to front-end bandwidth estimation, controlled errors were defined relative to the ground-truth occupied bandwidth rather than added to the model’s native estimate. For an error bound ε, the bandwidth supplied to the router was B(1 + δ), where B denotes the ground-truth bandwidth and δ was sampled uniformly from [−ε, ε]. The horizontal axis therefore represents the half-width of the admissible error interval rather than a fixed signed bias. Ordinary inference using the native detector output served as the baseline for both profile-change probability and output NMSE. Relative NMSE change (%) was calculated from linear NMSE values before conversion to decibels as 100 × [NMSE under error bound ε − baseline NMSE]/baseline NMSE, where baseline NMSE denotes ordinary detector-driven inference; negative and positive values indicate improvement and degradation, respectively. Consequently, ε = 0 is an oracle-bandwidth condition, not ordinary model inference; it can slightly improve NMSE because it removes the detector’s residual estimation error.

**Figure 12:**
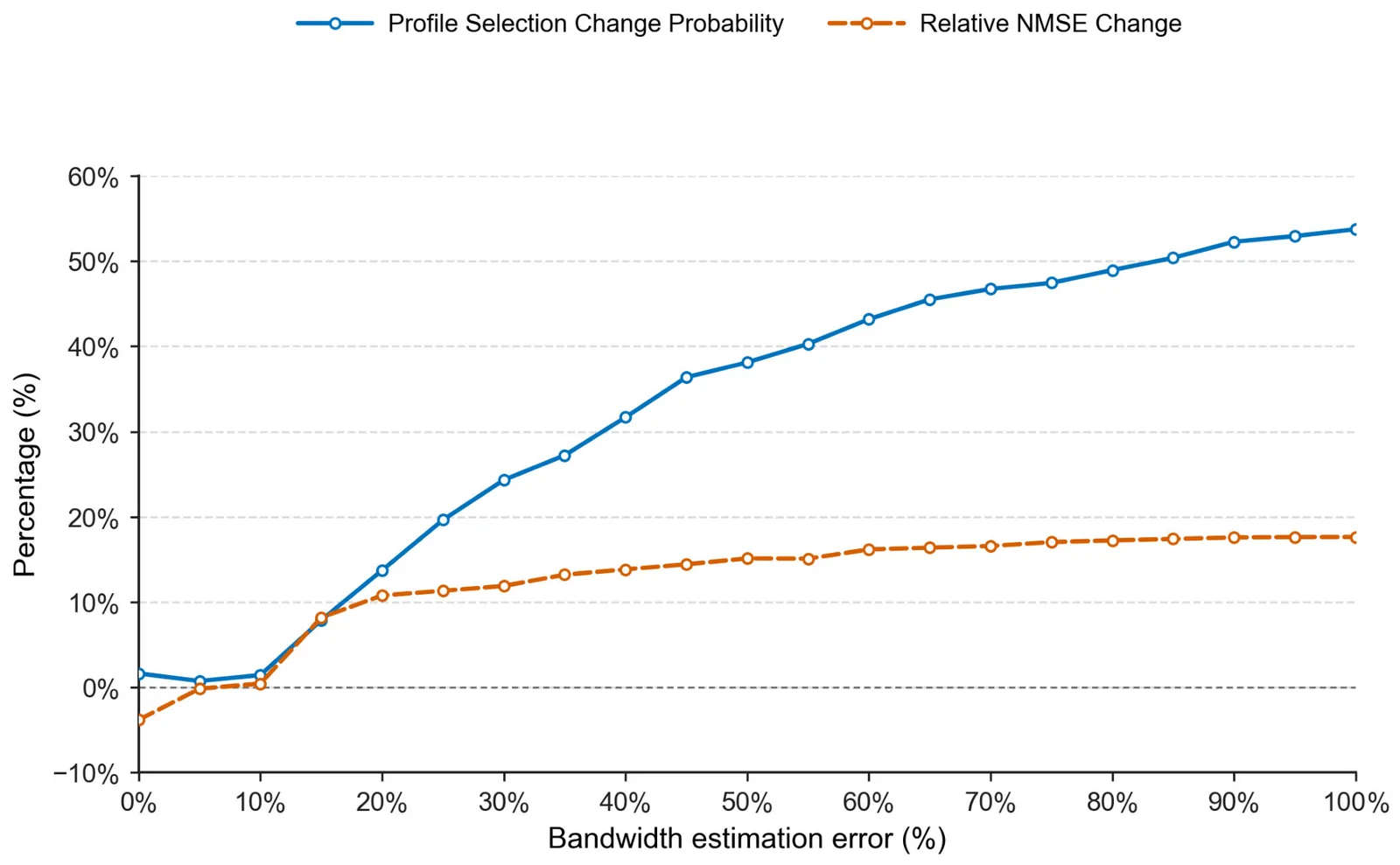
Sensitivity of adaptive-profile selection and output NMSE to controlled bandwidth errors referenced to the ground-truth occupied bandwidth.

As shown in Figure 12, at 0% controlled error, supplying the ground-truth bandwidth produces a small NMSE improvement relative to normal detector-driven inference and can alter a small fraction of profile choices. This reflects the residual error of the native bandwidth estimator. As the error bound increases, the probability of changing the selected profile rises monotonically and reaches approximately 54% at a 100% error bound, while the relative NMSE change remains below 18%. Crossing a routing boundary therefore produces only a modest change in reconstruction quality because adjacent feasible profiles share the same separator and overlapping receptive-field coverage. Together with the ablation results in Table 2, these findings indicate that adaptive routing remains beneficial across the tested bandwidth-error range.

### 4.6. Ablation and Mechanistic Interpretation

Removing adaptive sampling reduced mean SI-SDRi by 2.98, 4.44 and 4.47 dB on D1, D2 and D3, respectively. The larger degradation on D2 and D3 indicates that profile routing primarily improves robustness to acquisition-dependent scale mismatch by coordinating processing rate, convolutional scale and the physical receptive field of the shared separator.

Removing Mamba reduced mean SI-SDRi by 4.01, 2.22 and 2.36 dB on D1–D3 and increased log-PSD RMSE to 16.65, 17.74 and 17.26 dB, respectively. This result supports complementary roles for the two sequence operators: dilated convolutions capture local oscillations and multiscale neighbourhoods, while Mamba helps maintain source identity over longer intervals.

Removing FiLM while retaining the adaptive profile bank reduced the parameter count by 190,848, but increased mean NMSE by 10.87, 12.40 and 12.20 dB on D1–D3 and raised log-PSD RMSE by 2.26, 3.23 and 5.60 dB, respectively. The increasing spectral penalty on the cross-domain datasets indicates that continuous condition-dependent modulation complements discrete profile routing by adapting the shared separator to signal- and acquisition-specific variation.

Wave-U-Net and DPRNN are competitive under several conditions. However, Wave-U-Net loses more performance on the heterogeneous D3 signals, while DPRNN requires the largest memory footprint. The Transformer is competitive on D1 but degrades markedly on D2 and D3. Overall, these comparisons suggest that the Adaptive-Sampling Mamba–Conv-TasNet provides comparatively consistent cross-domain separation with moderate latency, model size and memory use.

## 5. Conclusions

This work introduces the Adaptive-Sampling Mamba–Conv-TasNet for separating two electromagnetic sources from a single complex-baseband observation. The central design principle is to align the discrete representation with the physical bandwidth and time scale of each input before applying a shared separator. Anti-aliasing-constrained profile routing and multiscale convolution provide this alignment, while the TCN–Mamba backbone combines local waveform modelling with selective long-range memory.

Among the nine evaluated methods, the Adaptive-Sampling Mamba–Conv-TasNet achieved the lowest mean complex NMSE on all three test sets and the lowest log-PSD RMSE on D2 and D3. It also maintained comparatively stable SI-SDRi across SNR levels. The ablations associate these gains with adaptive scale selection, FiLM conditioning and Mamba-based long-range modelling. Collectively, the results indicate a favourable overall trade-off among separation quality, computational efficiency and resource use under the evaluated acquisition conditions.

## Figures and Tables

**Table 1:** Parameters of the four adaptive profile encoders.

Profile	Encoder Stride	Kernel Lengths
P1	8	8, 16, 32, 64
P2	4	4, 8, 16, 32
P3	2	2, 4, 8, 16
P4	2	2, 4, 8, 16

**Table 2:** Computational efficiency, resource use and reconstruction errors on D1–D3 (mean ± standard deviation for reconstruction errors).

Dataset	Model	Latency (ms)	RTF	Trainable Parameters	Peak CUDA (MB)	Peak CPU (MB)	NMSE (dB)	Log-PSD RMSE (dB)
D1	Proposed	5.788	220.80	1,416,912	23.3984	1265.38	−25.08 ± 1.27	5.77 ± 0.94
	Transformer	7.03	268.00	1,331,104	23.9629	1283.60	−18.84 ± 3.68	5.74 ± 2.05
	Wave-U-Net	5.60	213.60	8,358,508	47.9805	1350.38	−18.79 ± 1.86	6.28 ± 1.42
	DPRNN	9.96	380.10	1,763,456	70.7495	1415.49	−21.47 ± 1.53	7.95 ± 1.73
	EEMD–ICA	60.05	2290.80	0	-	656.69	−4.28 ± 5.72	9.44 ± 0.80
	VMD–ICA	16.85	642.90	0	-	600.36	−2.03 ± 4.26	10.83 ± 1.22
	Ablation without Mamba	4.33	165.30	1,066,704	16.7349	1280.80	−14.74 ± 4.48	16.65 ± 1.92
	Ablation without adaptive sampling	5.35	204.00	1,290,564	21.9150	1281.48	−16.40 ± 2.93	6.43 ± 1.62
	Ablation without FiLM	4.84	184.50	1,226,064	22.2803	1264.33	−14.21 ± 3.43	8.03 ± 1.22
D2	Proposed	6.77	132.30	1,416,912	23.3984	1265.38	−22.66 ± 1.67	6.38 ± 1.48
	Transformer	23.83	465.50	1,331,104	23.9629	1283.60	−2.98 ± 1.59	16.51 ± 2.56
	Wave-U-Net	7.65	149.40	8,358,508	47.9805	1350.38	−13.64 ± 2.16	9.77 ± 3.79
	DPRNN	14.72	287.40	1,763,456	70.7495	1415.49	−17.20 ± 1.86	8.91 ± 2.01
	EEMD–ICA	71.53	1397.00	0	-	656.69	0.31 ± 7.26	23.39 ± 3.47
	VMD–ICA	29.20	570.30	0	-	600.36	−0.07 ± 6.73	19.62 ± 2.97
	Ablation without Mamba	5.28	103.20	1,066,704	16.7349	1280.80	−13.50 ± 2.27	17.74 ± 2.92
	Ablation without adaptive sampling	6.42	125.40	1,290,564	21.9150	1281.48	−8.34 ± 1.72	11.45 ± 1.89
	Ablation without FiLM	9.032	176.40	1,226,064	22.2803	1264.33	−10.26 ± 1.29	9.61 ± 1.39
D3	Proposed	6.07	74.10	1,416,912	23.3984	1265.38	−20.27 ± 3.58	6.26 ± 0.99
	Transformer	96.09	1173.00	1,331,104	23.9629	1283.60	−1.49 ± 3.87	18.17 ± 1.98
	Wave-U-Net	9.04	110.40	8,358,508	47.9805	1350.38	−8.26 ± 2.15	10.54 ± 1.87
	DPRNN	20.79	253.80	1,763,456	70.7495	1415.49	−11.64 ± 2.70	9.27 ± 2.81
	EEMD–ICA	99.43	1213.70	0	-	656.69	2.33 ± 3.86	25.76 ± 2.42
	VMD–ICA	47.41	578.70	0	-	600.36	1.88 ± 4.97	22.79 ± 2.67
	Ablation without Mamba	8.92	108.90	1,066,704	16.7349	1280.80	−13.43 ± 5.14	17.26 ± 2.63
	Ablation without adaptive sampling	6.00	73.20	1,290,564	21.9150	1281.48	−6.17 ± 5.38	13.09 ± 1.76
	Ablation without FiLM	14.57	177.90	1,226,064	22.2803	1264.33	−8.07 ± 3.13	11.86 ± 2.03

## Data Availability

The raw data supporting the conclusions of this article will be made available by the authors on request.

## Appendix A. Key Parameters of Data, Models, and Experimental Setup

This appendix summarises the parameters that materially affect data reproducibility, model architecture and training. Parameter counts cover offline-learned weights; run-time decomposition coefficients and demixing matrices of the conventional methods are excluded from that count.

### Appendix A.1. Training and Test Data

**Table A1:** Datasets, data formats, and purposes.

Dataset and Signal Category	Original Data Format	Sampling Rate	Centre Frequency	Remarks
MPACT Drone Remote Controller RF Signal Dataset: Inspire 1 Pro, Matrice 100, Matrice 600_1, and Matrice 600_2	MATLAB v5; signed sample codes calibrated and converted to float32	20 GHz	2.4 GHz	Training dataset containing 3289 training records and 822 validation records; recordings are isolated between splits
MPACT: DJI Phantom 3 and DJI Phantom 4 Pro	MATLAB v5; signed sample codes calibrated and converted to float32	20 GHz	2.4 GHz	Test dataset acquired using the same data-collection framework as the training data, but with transmitter models not used for training
DroneMMSet RF subset	Complex I/Q files (.iq)	40 MHz	2.4 GHz	Test dataset for cross-dataset and cross-device evaluation
MIT RF Challenge	SigMF cf32_le, complex64	25 MHz	2.4/5.8 GHz	Test dataset for evaluating transfer to communication signals and different acquisition conditions

**Table A2:** Mixture generation and evaluation protocol.

Item	Method or Parameter	Value
Sampling-rate conversion	Shared polyphase resampling with a Kaiser-window low-pass FIR	Selected profile rate F_p_; target stop-band attenuation of 90 dB; pass-band edge at 0.8 times the output Nyquist frequency for rate-reducing routes
Frequency alignment	Power-spectral-centroid estimation and common frequency shift	The same frequency shift is applied to the mixture, targets, and noise
Signal-to-interference ratio	Power scaling	SIR ~ Uniform (−5, 5) dB
Training segments	Stratified token-length sampling	Core lengths of 512, 1024, and 2048 tokens are sampled with probabilities 0.20, 0.65, and 0.15, respectively; equal-length context is added on both sides, giving a complete sequence three times the core length.
Noise type	Clean/synthetic/impulse/measured	Clean, synthetic, impulse, and measured noise are sampled with probabilities 0.35, 0.30, 0.05, and 0.30, respectively; white and coloured noise are equally represented within the synthetic category.
Training-noise intensity	Piecewise SNR sampling	Among noisy examples, the high (12–30 dB), medium (3–12 dB), and low (−2–3 dB) ranges are sampled with probabilities 0.65/0.30/0.05
Amplitude normalisation	Shared robust RMS after 0.999-quantile clipping	The mixture, both targets, and noise use the same scale factor
Data partitioning	Recording/group isolation; SHA-256 deduplication	Validation proportion = 20%.
Test-signal length	Complex I/Q sequence	2048 samples
Test SNR	Fixed grid	−10, −6, −2, 2, 6, 10, 14, 18, 22, 26, and 30 dB

**Table A3:** Processing rates and encoder parameters of the four adaptive profiles.

Profile	Processing Rate	Encoder Stride	Kernel Lengths
P1	312.5 MHz	8	(8,16,32,64)
P2	156.25 MHz	4	(4,8,16,32)
P3	78.125 MHz	2	(2,4,8,16)
P4	25 MHz	2	(2,4,8,16)

All four profiles use the same routing and processing flow. The router selects the lowest sampling-rate-compatible configuration that satisfies the occupied-band constraint, and the active encoder and decoder use the corresponding parameters in Table A3.

**Table A4:** Detector and routing parameters.

Parameter	Executed Definition or Value
Spectrum estimation	Mean power spectrum over Hann-windowed frames; 50% overlap.
FFT length	min(4096, 2 ^floor(log2(max(*L*, 64)))^), with a minimum of 64 samples.
Noise floor	20th percentile of the power spectrum expressed in decibels.
Occupied band	Central 95% and 99% cumulative-power intervals provide *B*_95_ and *B*_99_; routing uses the recentred *B*_99_ bounds.
Active clusters	Threshold = noise floor + 6 dB; at least 3 consecutive FFT bins; retain clusters with at least 1% of the strongest-cluster power.
Frequency recentring	Cluster-based spectral centre: power-weighted centroid for one retained cluster, midpoint of the outer retained edges for multiple clusters, and full-spectrum centroid if no cluster is retained; the same digital shift is applied to the mixture, targets, and noise.
Routing radius	r = max(|*f*_low_|, |*f*_high_|) after recentring.
Profile selection	Among sampling-rate-compatible profiles, select the lowest configured rate satisfying F_p_/2 ≥ ηr for rate-reducing routes; η = 1.25.
Sampling-rate conversion	Convert the centred waveform to the selected profile rate with the shared polyphase Kaiser-window FIR operator.
Detector confidence	0.35 clip(SNR/12) + 0.35 min(activity/0.6, 1) + 0.30 I(cluster), clipped to [0, 1].

**Table A5:** Ordered composition of the 26-dimensional condition vector.

Dimensions	Feature	Normalisation or Encoding
1	Processing rate *F_p_*	log_10_(*F_p_*)
2	95% occupied bandwidth *B*_95_	*B*_95_/*F_p_*
3	99% occupied bandwidth *B*_99_	*B*_99_/*F_p_*
4	Lower occupied-band edge	*f*_low_/*F_p_*
5	Upper occupied-band edge	*f*_high_/*F_p_*
6	Spectral-centroid correction	Recentring shift divided by *F_p_*
7–9	Three dominant time scales τ_1_–τ_3_	log_10_(max(τ_k_, 10^−12^))
10	Burst length	log_10_(max(value, 10^−12^))
11	Burst interval	log_10_(max(value, 10^−12^))
12	Detector SNR	SNR/30; not clipped
13	Spectral flatness	Unchanged
14	Nonstationarity	Frame-power standard deviation divided by its mean
15	Detector confidence	Clipped to [0, 1]
16	Occupied-band radius	r/(*F_p_*/2)
17	Profile index	Profile index divided by (number of profiles − 1): P1/P2/P3/P4 = 0, 1/3, 2/3, 1
18	Validity summary	Mean of the eight validity indicators
19–26	Validity indicators	isfinite indicators for *B*_95_, *B*_99_, *f*_low_, *f*_high_, centroid, SNR, flatness, and nonstationarity, in that order

Dominant lags are obtained from the normalised autocorrelation of the mean-centred blockwise envelope-energy sequence. Lag zero is excluded; up to three nonzero lags with autocorrelation greater than 0.1 are retained and converted to seconds as τ_q_ = ℓ_q_K/F_in_, where K is the block length in samples. Missing lags are padded with zero and encoded as −12 after the logarithmic transformation in Table A5.

### Appendix A.2. Model Architectures and Training Parameters

All learned models and ablation variants were trained for 16,000 parameter updates. The stage proportions and remaining optimisation settings are listed in the corresponding model tables.

**Table A6:** Adaptive-Sampling Mamba–Conv-TasNet.

Module	Architecture or Method	Key Parameters	Trainable Parameters
Physical-feature extraction and profile routing	Hann-windowed mean power spectrum, B_95_/B_99_, cluster-based spectral centre, envelope activity, and block-energy ACF dominant lag	FFT size ≤ 4096; 50% overlap; 20th-percentile noise floor; detection threshold +6 dB; 18-dimensional features plus an 8-dimensional validity vector; guard factor = 1.25	0
Adaptive analysis encoder	Four profile-specific encoders, each comprising four Conv1d(2→32) branches followed by concatenation, Conv1d(128→128,1), LayerNorm, and PReLU	Profile-specific strides and kernels are listed in Table A3; latent channels = 128	82,432
Conditioning encoder	Two-layer MLP (26→192→192) with SiLU activations and intermediate LayerNorm	—	42,624
FiLM conditioning	One fully connected layer (192→256) before each TCN repeat, producing 128-dimensional scale and bias vectors	Three independent, zero-initialised FiLM layers; output transformation: (1 + scale)x + bias	148,224
TCN local-separation module	3 repeats × 6 blocks with 1 × 1 bottleneck, depthwise dilated Conv1d, residual path, and skip path	Feature channels = 128; bottleneck channels = 96; kernel size = 3; dilation factors = 1, 2, 4, 8, 16, and 32; dropout = 0.05	679,104
Mamba long-range module	One independent Mamba block after each TCN repeat	Three blocks with forward/backward/forward directions; model dimension = 128; state dimension = 16; convolution width = 4; expansion factor = 2; time-step rank = 8	350,208
Mask head	PReLU + LayerNorm + Conv1d(128→256,1), reshaped into two 128-channel masks	One shared head; independent sigmoid masks	33,408
Adaptive synthesis decoder	Four profile-specific decoders, each comprising Conv1d(128→128,1) and four ConvTranspose1d(32→2) branches	Softmax-weighted combination of four branches; exact cropping according to encoder padding	80,912
Training configuration	AdamW; PIT composite loss; BF16; four-stage curriculum (Stages 1–4: 10%, 20%, 50%, and 20% of training, respectively)	Initial learning rate = 1 × 10^−4^; reduced to 1.5 × 10^−5^ in Stage 4; minimum learning rate = 1 × 10^−6^; β = (0.9, 0.98); weight decay = 1 × 10^−4^; gradient clipping = 5	—
Total	—	—	1,416,912

**Table A7:** Transformer comparison model.

Module	Architecture or Method	Key Parameters	Trainable Parameters
Analysis encoder	Conv1d(2→128, kernel 16, stride 8) + LayerNorm	Same pre-processed two-channel real-valued representation of the complex-baseband waveform as the other learned models	4352
Positional encoding	Sinusoidal positional encoding	Sinusoidal base = 10,000; no trainable parameters	0
Transformer encoder	Six-layer global bidirectional self-attention encoder	Model dimension = 128; 8 attention heads; head dimension = 16; feed-forward dimension = 512; dropout = 0.10; ReLU; post-norm	1,289,632
Mask head	Linear(128→256) + sigmoid	Two 128-channel masks	33,024
Synthesis decoder	ConvTranspose1d(128→2, kernel 16, stride 8)	Output cropped to the original length	4096
Training configuration	Same optimiser, PIT composite loss, BF16 precision, and four-stage curriculum (Stages 1–4: 10%, 20%, 50%, and 20% of training, respectively) as the main model	Initial learning rate = 1 × 10^−4^; reduced to 1.5 × 10^−5^ in Stage 4	—
Total	—	—	1,331,104

**Table A8:** Wave-U-Net comparison model.

Module	Architecture or Method	Key Parameters	Trainable Parameters
Input and length handling	Directly accepts the preprocessed complex-baseband waveform; right zero-padding extends the input to a multiple of 2^5^ = 32, and the output is cropped to the original length	Two-channel real-valued representation of the complex-baseband input	0
Five-level convolutional encoder	Each level contains two same-padded Conv1d layers, GroupNorm, and LeakyReLU; dropout follows the first activation; a Conv1d layer with kernel 4, stride 2, and padding 1 downsamples between levels	Channels = 24, 48, 96, 192, and 384; convolution kernel size = 5; dropout = 0.05	2,948,592
Bottleneck	Two Conv1d(384→384) layers with kernel size 5, GroupNorm, LeakyReLU, and dropout	Bottleneck channels = 384	1,476,864
Five-level convolutional decoder	A ConvTranspose1d layer with kernel 4, stride 2, and padding 1 upsamples the representation, which is concatenated with the corresponding encoder feature and processed by a double-convolution block	U-Net skip connections; channels restored in the order 384/192/96/48/24	3,932,952
Waveform output layer	Conv1d(24→4, kernel size 1), reshaped into two complex-baseband sources	Direct prediction of two complex-baseband source waveforms	100
Training configuration	AdamW; same PIT composite loss as the main model; BF16; four-stage curriculum (Stages 1–4: 10%, 20%, 50%, and 20% of training, respectively)	Initial learning rate = 1 × 10^−4^; reduced to 1.5 × 10^−5^ in Stage 4; β = (0.9, 0.98); weight decay = 1 × 10^−4^; gradient clipping = 5	—
Total	One-dimensional Wave-U-Net with depth = 5	Theoretical FP32-weight footprint of approximately 31.89 MiB	8,358,508

**Table A9:** DPRNN-TasNet comparison model.

Module	Architecture or Method	Key Parameters	Trainable Parameters
Profile-specific analysis/synthesis codec	Four bias-free Conv1d(2→128)/ConvTranspose1d(128→2) pairs; ReLU after encoding	P1–P4 use strides 8/4/2/2 and kernel lengths 16/8/4/4; latent channels = 128	16,384
Bottleneck	GroupNorm + Conv1d(128→64, kernel size 1)	Bottleneck channels = 64	8512
Dual-path segmentation	The encoded sequence is divided into 50–overlapping chunks and recombined by normalised overlap-add	Chunk length = 100; 50% overlap	0
DPRNN separation module	Four DPRNN blocks; each block applies intra-chunk and inter-chunk bidirectional LSTMs followed by linear projection, LayerNorm, dropout, and residual connection	LSTM input = 64; hidden size = 128; bidirectional intra- and inter-chunk LSTMs; dropout = 0.05; 430,464 parameters per block	1,721,856
Mask head	PReLU + Conv1d(64→256, kernel size 1), reshaped into two 128-channel masks	Independent sigmoid masks for the two sources	16,704
Source reconstruction	Both masks are applied to the same encoded representation; the current profile’s transposed-convolution decoder reconstructs each source	Two complex-baseband sources are reconstructed; output is cropped exactly according to codec padding	0
Training configuration	AdamW; same PIT composite loss as the main model; BF16; four-stage curriculum (Stages 1–4: 10%, 20%, 50%, and 20% of training, respectively)	Initial learning rate = 1 × 10^−4^; reduced to 1.5 × 10^−5^ in Stage 4; β = (0.9, 0.98); weight decay = 1 × 10^−4^; gradient clipping = 5	—
Total	Four-block DPRNN-TasNet	Theoretical FP32-weight footprint of approximately 6.73 MiB	1,763,456

**Table A10:** EEMD–ICA comparison method.

Module	Architecture or Method	Key Parameters	Trainable Parameters
EEMD decomposition	Noise-assisted EMD is applied separately to the I and Q components of the complex mixture, using cubic-spline upper and lower envelopes and Gaussian auxiliary noise with alternating signs across ensemble iterations	Ensemble size = 20; auxiliary-noise standard-deviation ratio = 0.20; at most 9 modes, including the final residual; at most 30 sifting iterations per IMF; sifting tolerance = 0.05	0
Complex pseudo-channel construction	I- and Q-component decompositions are paired by IMF order to form complex pseudo-channels	Nine complex pseudo-channels; residual correction ensures that the modes sum to the input	0
FastICA demixing	After centring the complex pseudo-channels, the real and imaginary parts are concatenated along the observation dimension; PCA whitening and symmetric FastICA are then applied, and the same real-valued demixing matrix is used for the complex pseudo-channels	Two output components; tanh nonlinearity; at most 100 ICA iterations; convergence tolerance = 1 × 10^−4^	0
Complex-scale restoration and output	Least-squares projection-back restores the complex amplitude and phase relative to the observed mixture and produces two complex-baseband sources	One complex-baseband mixture is mapped to two complex-baseband source estimates	0
Total	Per-signal decomposition and demixing	Decomposition results and the ICA demixing matrix are estimated independently for each signal	0

**Table A11:** VMD–ICA comparison method.

Module	Architecture or Method	Key Parameters	Trainable Parameters
VMD decomposition	Mirror extension and variational mode decomposition are applied separately to the I and Q components of the complex mixture	Three modes; bandwidth penalty = 2000; noise-tolerance parameter = 0; DC mode not fixed; uniformly initialised centre frequencies; convergence tolerance = 1 × 10^−7^; at most 500 VMD iterations	0
Complex pseudo-channel construction	I and Q modes are paired in initialisation order to form complex pseudo-channels	Three complex pseudo-channels; residual correction of the last mode ensures that the modes sum to the input	0
FastICA demixing	Uses the same centring, whitening, symmetric FastICA, and complex-valued demixing procedure as EEMD–ICA	Two output components; tanh nonlinearity; at most 100 ICA iterations; convergence tolerance = 1 × 10^−4^	0
Complex-scale restoration and output	Least-squares projection-back produces two complex-baseband sources	One complex-baseband mixture is mapped to two complex-baseband source estimates	0
Total	Per-signal decomposition and demixing	VMD modes and the ICA demixing matrix are estimated independently for each signal	0

**Table A12:** Ablation model without Mamba.

Module	Architecture or Method	Key Parameters	Trainable Parameters
Physical-feature extraction and profile routing	Same as the main model	18-dimensional features plus 8-dimensional validity vector; four adaptive profiles	0
Adaptive analysis encoder and synthesis decoder	Same four-profile encoder and decoder as the main model	Encoder 82,432; decoder 80,912	163,344
Conditioning MLP + FiLM	Same as the main model	MLP 26→192→192; three FiLM layers	190,848
TCN main separation module	Same as the main model	3 repeats × 6 blocks; dilation = (1,2,4,8,16,32)	679,104
Mamba	Mamba blocks omitted	—	0
Mask head	Same as the main model	Conv1d(128→256,1) + sigmoid	33,408
Training configuration	Same optimiser, PIT composite loss, BF16 precision, and four-stage curriculum (Stages 1–4: 10%, 20%, 50%, and 20% of training, respectively) as the main model	Same learning-rate schedule and regularisation settings as the main model	—
Total	—	—	1,066,704

**Table A13:** Ablation model without adaptive sampling.

Module	Architecture or Method	Key Parameters	Trainable Parameters
Detection and preprocessing	Physical-feature detection, spectral-centroid correction, and anti-aliasing FIR are retained, whereas adaptive profile routing is disabled	All inputs are processed at 25 MHz	0
Fixed multiscale analysis encoder	One four-branch encoder	Stride = 2; kernels = (2,4,8,16); channels = 128	18,688
Conditioning MLP + FiLM	Same as the main model	MLP 26→192→192; three FiLM layers; one fixed conditioning profile	190,848
TCN main separation module	Same as the main model	3 repeats × 6 blocks	679,104
Mamba long-range module	Same as the main model	Three blocks with forward/backward/forward directions; model dimension = 128; state dimension = 16; convolution width = 4; expansion factor = 2	350,208
Mask head	Same as the main model	Conv1d(128→256,1) + sigmoid	33,408
Fixed synthesis decoder	One four-branch decoder	stride = 2; kernels = (2,4,8,16)	18,308
Training configuration	Same optimiser, PIT composite loss, BF16 precision, and four-stage curriculum (Stages 1–4: 10%, 20%, 50%, and 20% of training, respectively) as the main model	Same learning-rate schedule and regularisation settings as the main model	—
Total	—	All parameters are trainable	1,290,564

**Table A14:** Ablation model without FiLM conditioning.

Module	Architecture or Method	Key Parameters	Trainable Parameters
Physical-feature extraction and profile routing	Same as the main model; bandwidth detection, spectral-centroid correction, anti-aliasing filtering, and four discrete profiles are retained	FFT size ≤ 4096; 50% overlap; 20th-percentile noise floor; detection threshold +6 dB; guard factor = 1.25; the lowest feasible processing rate is selected from the detected bandwidth	0
Adaptive analysis encoder and synthesis decoder	Same four-profile encoder and decoder as the main model	Encoder: 82,432; decoder: 80,912; profile-specific strides and kernels are listed in Table A3	163,344
Conditioning MLP	Conditioning MLP omitted	Main-model mapping 26→192→192 omitted	0
FiLM conditioning	Identity mapping before each TCN repeat	Separator transformation: y = x	0
TCN main separation module	Same as the main model	3 repeats × 6 blocks; feature channels = 128; bottleneck channels = 96; kernel size = 3; dilation factors = 1, 2, 4, 8, 16, and 32; dropout = 0.05	679,104
Mamba long-range module	Same as the main model	Three independent blocks with forward/backward/forward directions; model dimension = 128; state dimension = 16; convolution width = 4; expansion factor = 2; time-step rank = 8	350,208
Mask head	Same as the main model	PReLU + LayerNorm + Conv1d(128→256,1), reshaped into two 128-channel sigmoid masks	33,408
Training configuration	Same optimiser, PIT composite loss, BF16 precision, and four-stage curriculum (Stages 1–4: 10%, 20%, 50%, and 20% of training, respectively) as the main model; trained from random initialisation	Same learning-rate schedule and regularisation settings as the main model	—
Total	Adaptive sampling and discrete profile routing with identity conditioning	Removes 42,624 conditioning-MLP parameters and 148,224 FiLM parameters, for a total reduction of 190,848 parameters	1,226,064

### Appendix A.3. Hardware and Software Environment

**Table A15:** Experimental platform and key software versions.

Category	Item	Configuration/Version
Computer	Model	Lenovo Legion Y9000P IRX9 (machine type 83DF)
Computer	CPU	Intel Core i9-14900HX, 24 cores/32 threads
Computer	Memory	32 GB (approximately 31.7 GiB visible to the system)
Computer	GPU	NVIDIA GeForce RTX 4060 Laptop GPU, 8188 MiB VRAM
Operating system	Linux	Ubuntu 22.04.5 LTS on WSL2
Runtime	Python	3.10.12
Deep learning	PyTorch	2.7.0+cu126
GPU software stack	CUDA/cuDNN	12.6/9.5.1
State-space model	mamba_ssm	2.3.2.post1
Convolution acceleration	causal_conv1d	1.6.2.post1
Numerical computing	NumPy/SciPy	2.2.6/1.15.3
Configuration parsing	PyYAML	6.0.3
